# Intrinsic Effects of Sulfidation on the Reactivity of Zero-Valent Iron With Trichloroethene: A DFT Study

**DOI:** 10.1021/acs.jpcc.3c04459

**Published:** 2023-10-24

**Authors:** Miroslav Brumovský, Daniel Tunega

**Affiliations:** † University of Natural Resources and Life Sciences, Vienna, Department of Forest- and Soil Sciences, Institute of Soil Research, Peter-Jordan-Straße 82, 1190 Vienna, Austria

## Abstract

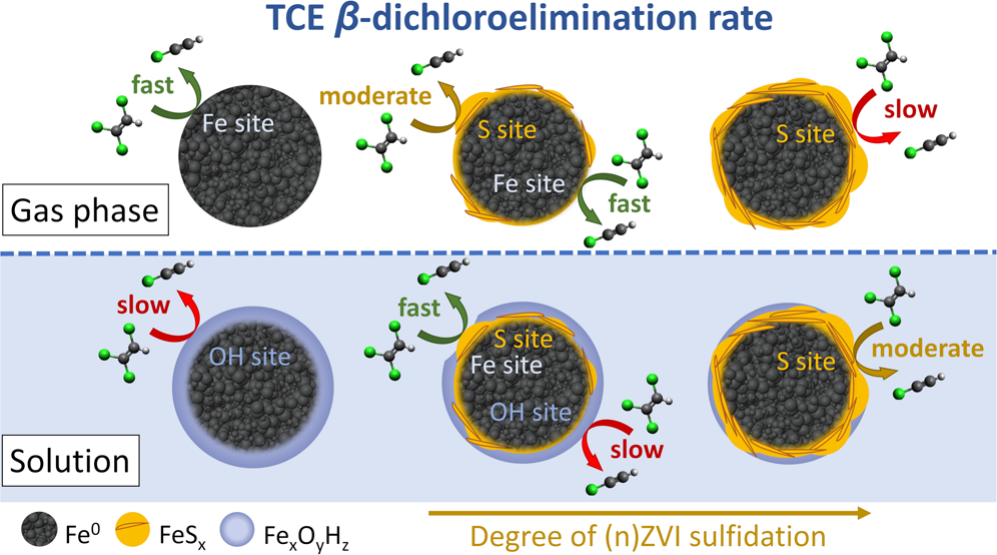

Sulfidation represents a promising approach to enhance the selectivity and longevity of zero-valent iron (ZVI) in water treatment, particularly for nanoscale ZVI (nZVI). While previous mechanistic studies have primarily concentrated on the impact of sulfidation on the (n)ZVI hydrophobicity, the fundamental effects of sulfidation on the (n)ZVI reactivity with target contaminants remain poorly understood. Herein, we employed density functional theory to elucidate reaction mechanisms of trichloroethene (TCE) dechlorination at various (n)ZVI surface models, ranging from pristine Fe^0^ to regularly sulfidated Fe surfaces. Our findings indicate that sulfidation intrinsically hinders the TCE dechlorination by (n)ZVI, which aligns with prior observations of sulfur poisoning in transition metal catalysts. We further demonstrate that the positive effects of sulfidation emerge when the surface of (n)ZVI undergoes corrosion. Notably, S sites exhibit higher reactivity compared to the sites typically present on the surface of (n)ZVI oxidized in water. Additionally, S sites protect nearby Fe sites against oxidation and make them more selective for direct electron transfer. Overall, our results reveal that the reactivity of sulfidated (n)ZVI is governed by an interplay of intrinsic inhibitory effects and corrosion protection. A deeper understanding of these phenomena may provide new insights into the selectivity of sulfidated (n)ZVI for specific contaminants.

## 1 Introduction

Zero-valent iron (ZVI) is a widely used material for in situ chemical reduction and sequestration of groundwater contaminants, such as chlorinated solvents, nitroaromatic compounds, heavy metals, and radionuclides.^1−4^ The nanoscale form of ZVI (nZVI) exhibits substantially higher reactivity compared to bulkier iron materials due to its large specific surface area. This enables nZVI to efficiently degrade a broader range of contaminants and limits the formation of undesirable intermediates.^5,6^ Yet, pristine nZVI is also very reactive with naturally occurring reducible species, such as water and oxygen, resulting in rapid corrosion and short longevity of nZVI in the aquifer.^7−9^ This leads to poor overall contaminant removal efficiency with nZVI and increased treatment costs.

In the past decade, sulfidation has been extensively studied as a promising approach to improve the selectivity of (n)ZVI for target contaminants.^10,11^ Two synthesis methods were typically used to prepare sulfidated nZVI (S-nZVI), consisting either of adding a sulfidation agent (e.g., Na_2_S or Na_2_S_2_O_4_) into a suspension of presynthesized nZVI particles or concurrent formation of mixed-phase Fe/FeS_*x*_/S nanoparticles (e.g., by adding NaBH_4_ and Na_2_S/Na_2_S_2_O_4_ into a solution containing Fe^3+^/Fe^2+^ ions).^10,11^ These processes have been referred to as “postsulfidation” and “one-pot.”^11^ Sulfidation of larger (microscale) ZVI particles has been performed by the postsulfidation method or by ball milling of ZVI with sulfur.^11^ Despite differences in morphology, sulfur content, and speciation, S-(n)ZVI prepared by all the above methods was found to possess higher reactivity with many contaminants, especially the chlorinated solvent trichloroethene (TCE)^12−17^ and substantially slower corrosion rate compared to pristine (n)ZVI, resulting in a longer reactive lifetime.^13,15,17,18^ Soon after the publication of the first experimental studies reporting the advantages of (n)ZVI sulfidation, research focused on investigating the mechanisms responsible for the observed reactivity and selectivity improvements. These include (a) higher hydrophobicity of the S-(n)ZVI surface, leading to the preferential sorption of hydrophobic contaminants in aqueous environments and lower surface affinity for water,^16,19−21^ (b) slower corrosion due to the suppresion of water adsorption and recombination of atomic hydrogen (H*) at the S-(n)ZVI surface,^13,19,22^ (c) a more porous structure of the corrosion products formed on S-(n)ZVI that does not lead to the surface passivation in contrast to the compact (oxyhydr)oxide layer formed on (n)ZVI,^21^ and (d) easier electron transfer across FeS_*x*_ phases compared to the oxidation products that typically occur on the surface of (n)ZVI.^19,22−24^ Despite these advances, it is not clear whether sulfidation also intrinsically enhances the (n)ZVI reactivity with target contaminants such as chlorinated solvents (e.g., by providing reactive catalytic sites) or whether their faster removal is indirectly caused by the lower S-(n)ZVI affinity for water that suppresses competing corrosion reactions and promotes reactions with target contaminants. Such knowledge is essential to understanding the mechanisms governing the performance and selectivity of S-(n)ZVI and may have major implications for the tailored design of S-(n)ZVI materials for specific environmental applications.

Molecular modeling based on quantum chemistry can provide important atomic-scale information about the electronic structure of surfaces of solid materials and their interactions with molecules. Moreover, the parameters of the modeled systems can be easily controlled (e.g., by creating specific surface defects and adding/removing solvent molecules), allowing for a better understanding of the effects of individual system components. Some of the studies dealing with the mechanistic aspects of S-(n)ZVI used theoretical approaches based on the density functional theory (DFT) to investigate the origin of increased hydrophobicity of S-(n)ZVI and the corrosion inhibition by iron sulfide minerals.^19−22^ However, the direct effects of sulfidation on (n)ZVI reactivity with target contaminants at the atomic scale are virtually unexplored. The authors are aware of only one study that investigated the reactivity of S-nZVI with the emerging contaminant florfenicol by using the DFT approach.^25^ In that study, Cao et al. reported adsorption configurations and DFT-calculated energies of florfenicol at the pristine and sulfidated Fe(110) surface and described how sulfidation changed the primary dechlorination pathway. Nevertheless, the authors did not calculate either the corresponding dehalogenation pathways or charge transfer to the florfenicol molecule. Despite the widespread application of S-(n)ZVI materials for the treatment of chlorinated solvents, a comprehensive understanding of the fundamental effects of sulfidation on their dechlorination during (n)ZVI-based treatments is still lacking.

This study aimed to systematically explore the intrinsic effects of sulfidation on the reactivity of (n)ZVI using TCE as a model halogenated contaminant. For this purpose, we calculated barriers for the electron transfer-controlled dechlorination reactions of TCE at various surface sites using the DFT approach. Multiple surface models were developed, particularly (a) pristine crystalline bcc *α*-Fe, (b) iron surface covered to various extent with S atoms, and (c) the same iron surface doped with an O atom/OH group/H*, enabling a deeper understanding of the role of sulfidation in the context of particle corrosion in aqueous environments. The computational results were interpreted alongside previously published experimental data. This study provides novel insights into the reactivity of S-(n)ZVI surfaces with halogenated contaminants at the atomic resolution.

## 2 Computational Details

### 2.1 Methods

The adsorption and dechlorination of TCE at various Fe-bearing surfaces were studied using spin-polarized plane-wave DFT calculations in periodic boundary conditions as implemented in the Vienna Ab initio Simulation Package (VASP).^26−28^ The electronic exchange–correlation potential was described using the generalized gradient approximation (GGA) with the Perdew–Burke–Ernzerhof (PBE) functional.^29^ The projector-augmented wave (PAW) method^30,31^ was used to describe the core–valence interaction. Long-range dispersion forces were accounted for through the DFT-D3 approach with the Becke–Johnson damping function.^32,33^ The convergence condition of the electronic self-consistent loop was set to 10^–6^ eV. Structural relaxation was achieved when the residual forces on all atoms were lower than 0.01 eV Å^–1^. The kinetic energy cutoff for plane waves in all calculations was 400 eV. The Brillouin zone was sampled using a 2 × 2 × 1 Monkhorst–Pack k-point mesh.^34^ Profiles of TCE dechlorination reactions were computed with the climbing image-nudged elastic band (CI-NEB) method.^35^ Solvation effects were included in the calculations using the continuum solvation model VASPsol^36,37^ on the gas-phase-optimized geometries. Further computational details are provided in Text S1 in the Supporting Information.

### 2.2 Modeled Systems

#### 2.2.1 Pristine Fe Surfaces

Periodic slab models representing the thermodynamically favorable (110) and (111) facets of pristine *α*-Fe were used for the calculation of the TCE adsorption energy and dechlorination barriers. The Fe(110) facet is the *α*-Fe closest-packed surface with the lowest surface energy.^38^ The Fe(111) facet is more open and rougher and hence was suggested to provide a better representation of the nZVI surface.^39^ Both slab models were constructed in our previous works^40,41^ from the fully relaxed *α*-Fe bulk structure. The Fe(110) slab model consisted of a 4 × 5 supercell with three atomic planes parallel to the surface. To validate the suitability of the 3-layer Fe(110) model, the TCE adsorption energy (Δ*E*_ads_) was calculated also with an analogical 7-layer Fe(110) model. The difference in Δ*E*_ads_ between the 3-layer and 7-layer models was <5 kJ mol^–1^. The Fe(111) slab model consisted of a 2 × 4 supercell with Fe atoms arranged in 7 layers.

#### 2.2.2 Fe Surfaces Doped with S

The effect of incorporation of S atoms at the pristine Fe surface on the TCE dechlorination was studied using four slab models with various numbers and configurations of S atoms: (a) with one S atom replacing one Fe surface atom, further referred to as “S-in-Fe(110)” according to the previous literature,^20^ (b) with one S atom bridging two Fe atoms at the hollow site of the Fe(110) surface, referred to as “S-on-Fe(110),” and (c) the Fe(110) surface covered with several S atoms on the hollow sites in a regular fashion, representing 1/4 and 1/2 monolayer coverage, referred to as “S_1/4 ML_–Fe(110)” and “S_1/2 ML_–Fe(110)”, with the topology of S atoms of the latter corresponding to the topmost layer of the (001) plane of mackinawite. The S-in-Fe(110) and S-on-Fe(110) models were based on the 3-layer Fe(110) surface slab described above. The slabs with higher S surface coverage were constructed from the optimized Fe bulk structure^40^ with 3-layer 4 × 4 Fe supercells, and their lateral lattice dimensions were relaxed after the surface was doped with S atoms.

#### 2.2.3 Fe Surfaces Doped with O, OH, and H

Slab models of Fe surfaces with one preadsorbed O atom (“O-on-Fe(110)”), OH group (“OH-on-Fe(110)”), and H atom (“H-on-Fe(110)”) were constructed in the same manner as the S-on-Fe(110) model. The surfaces doped with the O atom and the OH group allowed us to compare the effects of surface sulfidation and corrosion on the adsorption and dechlorination of TCE. Attempts to relax models containing O/OH within the Fe(110) surface (“O-in-Fe(110)” and “OH-in-Fe(110)”) resulted in surface reconstruction toward the “on” configurations, and, therefore, were not further considered. The Fe surface with preadsorbed H* was used to assess the effects of adsorbed H* originating from water dissociation on the electron transfer-controlled dechlorination of TCE.

Structures of all slab models are shown in Figure S1, together with their lattice parameters (Table S1). A 25 Å-thick vacuum layer was included in the direction perpendicular to the surface to decouple adjacent slabs. All surface models exhibited a ferromagnetic state, typical of bulk *α*-Fe.

## 3 Results and Discussion

### 3.1 Pristine ZVI is Extremely Reactive in the Dechlorination of TCE

**Figure 1:**
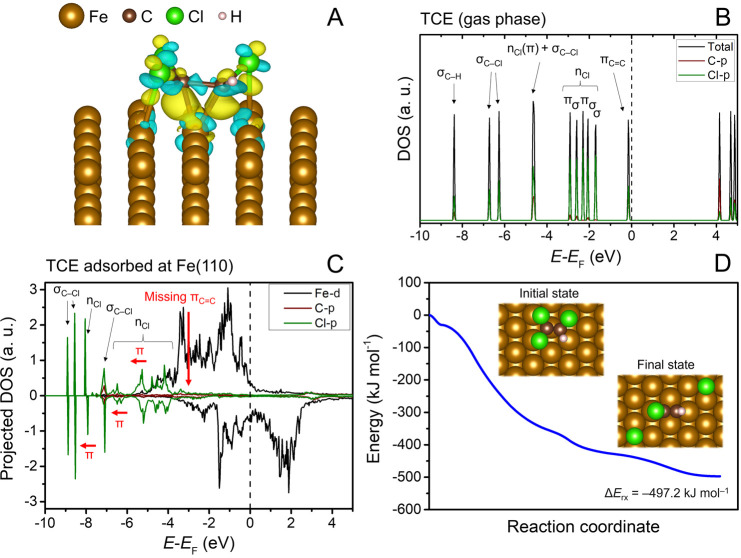
Adsorption and dechlorination of TCE at the pristine Fe(110) surface: (A) charge density difference plot of adsorbed TCE, (B) total and projected density of states (DOS) of an isolated TCE molecule, (C) projected DOS of adsorbed TCE with the main differences from the gas-phase molecule indicated in red, and (D) the reaction profile of TCE dechlorination with insets showing the calculated geometries. The yellow isosurface in panel (A) indicates an electron gain, while the blue isosurface represents an electron loss. The isosurface level was set to 0.005 with values in Bohr^–3^. The assignment of TCE molecular orbitals in DOS plots is according to Khvostenko.^47^ The initial state in panel (D) was calculated with fixed C–Cl distances to prevent spontaneous cleavage of Cl atoms.

The adsorption and dechlorination of TCE at the pristine Fe(110) surface were studied as a reference for further calculations. As shown in our recent study, full structural relaxations of TCE positioned in different orientations ca. 5 Å above the Fe(110) surface always led to the cleavage of two C–Cl bonds, corresponding to a spontaneous *β*-elimination reaction.^40^ The *β*-dichloroelimination was proposed to be the dominant TCE reduction pathway at the pristine (n)ZVI surface, followed by hydrogenolysis.^42^ To obtain the energy profile along the TCE dechlorination pathway, we optimized the TCE adsorption complex with all C–Cl bonds fixed to their length in the gas phase (Table S2) using the GADGET code.^43^ The TCE molecule adsorbed preferentially via the π_C=C_ bond at the atop Fe site (Figure S2), with the adsorption energy (Δ*E*_ads_) of −165.6 kJ mol^–1^. The length of the C=C bond notably increased from the TCE gas-phase geometry by 0.14 Å. The planar geometry of the isolated TCE molecule was strongly deformed upon adsorption, with the Cl–C–C–Cl and Cl–C–C–H dihedral angles decreasing from 180.0 to 130.8 and 133.0°, respectively (Table S2). The geometry distortion of TCE was accompanied by an increase in the charge density between the C=C bond and the Fe site (Figure 1A), indicating the formation of C–Fe bonds. This is in line with the relatively small distance between the C atoms and the Fe site in the adsorption complex of 1.95 and 2.05 Å (Figure S2).

The electronic density of states (DOS) plots illustrate the nature of the TCE interactions with the Fe site (Figure 1B,1C). The highest occupied molecular orbitals of TCE hybridize with Fe 3d orbitals, resulting in a broadening of their bands. The p_*z*_ orbitals of C and Cl, which are perpendicular to the TCE molecular plane, interact particularly strongly with the Fe site, leading to a complete disappearance of the π_C=C_ bond and stabilization of π nonbonding Cl orbitals. Our results agree with the previously proposed mechanism of TCE dechlorination at Fe surfaces, in which the weakening of the π_C=C_ bond and a concurrent formation of two σ_C–Fe_ bonds (i.e., partial sp^3^ hybridization of C atoms) lead to a strong TCE activation toward dechlorination reactions.^40,42,44−46^

Even with the reaction partitioned into 11 images, the CI-NEB calculation did not reveal any observable barrier for the TCE *β*-elimination reaction at the pristine Fe(110) surface (Figure 1D). This suggests that the activation energy for TCE dechlorination at this surface is negligible. The reaction products migrated to the hollow sites of the Fe(110) surface, and the reaction released energy of 497.2 kJ mol^–1^.

To provide an additional reference, the TCE dechlorination pathway at the pristine Fe(111) surface was also investigated. Again, cleavage of chlorine atoms from the TCE molecule during unconstrained structural relaxations of adsorption complexes typically occurred (with 3 initial configurations), yet one calculation converged with nondissociated TCE (Figure S2). In this configuration, adsorbed TCE was located over a hollow site between three top Fe atoms that interacted with Cl atoms, limiting the interaction of the C=C bond with underlying Fe atoms. Consequently, the Fe–C distances were ∼50% higher than in the TCE adsorption complex at the Fe(110) surface, and the adsorption was less favorable, with an Δ*E*_ads_ of only −59.7 kJ mol^–1^. The C=C and C–Cl bond lengths were slightly elongated compared to the TCE gas-phase geometry (Table S2), implying their weakening upon adsorption. Our efforts to fully optimize the geometry of one cleaved C–Cl bond always yielded a completely dechlorinated product with all species adsorbed at hollow sites. Even though the adsorbed TCE molecule was not noticeably activated for dechlorination reactions, the CI-NEB calculation showed only a small barrier of 8.8 kJ mol^–1^ (Figure S3). The complete TCE dechlorination released an energy of 708.6 kJ mol^–1^. These results further suggest that the dechlorination of TCE is favored at pristine Fe surfaces and occurs rapidly with a negligible activation barrier.

The spontaneous dissociation of TCE at Fe surfaces, as observed in the structural relaxations, is in agreement with previous DFT studies,^40,41,48^ as well as experimental studies performed by Auger electron spectroscopy, temperature-programmed desorption, and photoelectron spectroscopic methods under ultrahigh vacuum.^49,50^ Quasi-nondissociated TCE adsorption complexes at the Fe(110) surface were previously calculated using DFT only with a lax force convergence criterion^44^ of 0.514 eV Å^–1^. The reaction barrier for the first rate-limiting C–Cl cleavage was subsequently calculated by freezing all but the one dissociating Cl atom in the calculation^45^ and reached a relatively low value of 16.6 kJ mol^–1^. Altogether, these results demonstrate that Fe is extremely reactive with TCE and other chlorinated ethenes provided that its surface is pristine and not passivated, e.g., by oxidation products.

### 3.2 Doping of ZVI Surface with Sulfur Intrinsically Inhibits TCE Dechlorination

To study the intrinsic role of sulfur in the dechlorination of TCE by S-(n)ZVI particles, we first employed models of the Fe(110) surface doped with a single S atom, either replacing one Fe surface atom (“S-in-Fe(110)” model) or adsorbed at the surface hollow site (“S-on-Fe(110)” model). Such models were previously used to showcase the effect of (n)ZVI sulfidation on the blocking of water and H* adsorption.^20,22,51^

In the optimized geometry, TCE adsorbed at the S site of S-in-Fe(110) in a horizontal configuration with the C=C bond positioned ∼3.3 Å above the S atom (Figure S4). At the S site of S-on-Fe(110), TCE relaxed into a tilted configuration, with two Cl atoms pointing toward the slab surface (Figure S4). The distances between the C atoms and the S atom were ∼3.4 Å. In both adsorption complexes, the planar TCE geometry was only slightly distorted and resembled the structure in the gas phase (Table S2). No substantial C=C bond elongation or decrease in dihedral angles was apparent, implying low TCE activation for dechlorination reactions. The Δ*E*_ads_ of TCE at the S-in-Fe(110) and S-on-Fe(110) surfaces were of −81.8 and −62.8 kJ mol^–1^, respectively, being less favorable than at the pristine Fe(110) surface. The less favorable adsorption of TCE at the S-on-Fe(110) compared to the S-in-Fe(110) surface is due to the steric hindrance of the adsorbed S atom, which weakens the interaction between TCE and the iron surface, as discussed below.

**Figure 2:**
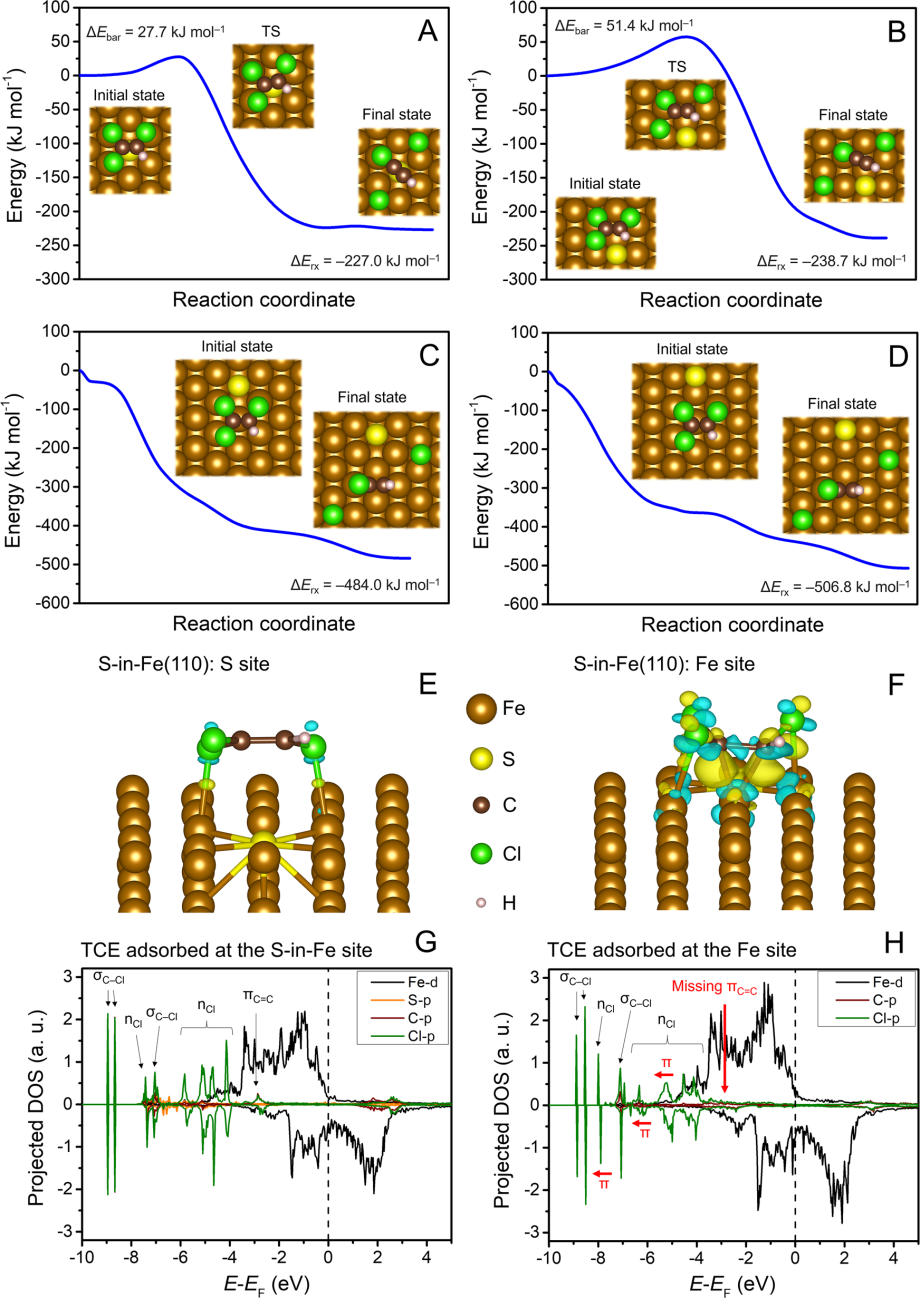
Adsorption and dechlorination of TCE at the Fe(110) surface doped with one S atom: reaction profiles of TCE dechlorination at the (A) S site of S-in-Fe(110), (B) S site of S-on-Fe(110), (C) Fe site of S-in-Fe(110), and (D) Fe site of S-on-Fe(110); (E) charge density difference plot of adsorbed TCE at the S site of S-in-Fe(110), (F) charge density difference plot of adsorbed TCE at the Fe site of S-in-Fe(110), (G) projected DOS of adsorbed TCE at the S site of S-in-Fe(110), and (H) projected DOS of adsorbed TCE at the Fe site of S-in-Fe(110) with the main differences indicated in red. In panels (A–D), insets show the calculated geometries, and TS denotes the transition state. The initial states in panels (C, D) were calculated with fixed C–Cl distances to prevent spontaneous cleavage of Cl atoms. The yellow isosurface in (E, F) indicates an electron gain, while the blue isosurface represents an electron loss. The isosurface level was set to 0.005 with values in Bohr^–3^. The assignment of TCE molecular orbitals in DOS plots is according to Khvostenko.^47^

Initially, we wanted to explore the energy profiles of sequential C–Cl bond cleavages as performed in our recent studies dealing with TCE dechlorination at the iron nitride surface.^40,46^ Accordingly, cleavage of the C–Cl bond with the lowest bond dissociation energy should yield *cis*-1,2-dichloroethene and Cl radicals.^45,46^ However, geometry optimizations of such dechlorination products at the S-doped surfaces always resulted in the spontaneous detachment of a second Cl atom from a vicinal C atom in the *trans* configuration, corresponding to a *trans*-*β*-elimination reaction (Figure 2A,2B). This mechanism aligns with the prior literature that suggested *β*-dichloroelimination to be the dominant TCE reduction pathway at S-(n)ZVI surfaces,^15,16,23,52^ with *trans*-stereochemistry being more favorable than *cis*.^53^ The calculated TCE *β*-elimination barriers were 27.7 and 51.4 kJ mol^–1^ at the S-in-Fe(110) and S-on-Fe(110) surfaces, respectively, i.e., at least twice higher than the barrier predicted at the Fe(110) surface by Lim et Lastoskie^45^ and three times higher than the barrier calculated at the pristine Fe(111) surface in this study. These results indicate that sulfidation of (n)ZVI intrinsically inhibits the dechlorination of TCE, with more significant inhibition occurring when the S atoms are adsorbed on the surface of pristine Fe compared with S atoms replacing Fe atoms in the topmost surface layer. TCE *β*-elimination reactions at S-in-Fe(110) and S-on-Fe(110) surfaces released energy of 227.0 and 238.7 kJ mol^–1^, respectively, which is about half of the energy released during TCE *β*-dichloroelimination at the Fe(110) surface. Given that the cleaved Cl atoms migrated to the hollow Fe sites just as in the reaction at the pristine Fe(110) surface, this large difference in reaction energies can be attributed to a less favorable adsorption configuration of chloroacetylene, which remained at a distance of ∼3.5 Å from the S atom at both S-doped surfaces.

To investigate whether the doping of the Fe surface with an S atom affects the activity of nearby Fe sites, we also evaluated the TCE adsorption and *β*-elimination at Fe sites adjacent to the S atoms. In the case of S-in-Fe(110), the nearest Fe atom to the S atom was chosen, while for S-on-Fe(110), a more distant Fe atom was chosen to avoid steric repulsion between the S atom and the TCE molecule. Similarly to the pristine Fe(110) surface, TCE underwent spontaneous dechlorination during structural relaxations at Fe sites of both S-in-Fe(110) and S-on-Fe(110) surfaces. After constraining the C–Cl bond lengths to their gas-phase geometry, the optimized adsorption complexes of TCE at the Fe sites were remarkably similar to those at pristine Fe(110) (Figure S5 and Table S2), with Δ*E*_ads_ reaching −166.1 and −158.8 kJ mol^–1^ at the S-in-Fe(110) and S-on-Fe(110) surfaces, respectively. No barriers were found in the CI-NEB calculations (Figure 2C,2D), and the reactions released energies of 484.0 and 506.8 kJ mol^–1^, respectively. The striking similarity between the TCE dechlorination profile at the Fe(110) surface and the Fe sites of S-doped Fe(110) surfaces implies that the doping of isolated S atoms to the Fe surface has a negligible effect on the reactivity of nearby accessible Fe sites in dehalogenation reactions and that TCE dechlorination is more favored at the Fe sites than at the S sites.

Cao et al. hypothesized that the S sites on the S-(n)ZVI surface are more reactive than Fe sites for direct electron transfer owing to charge redistribution toward the more electronegative S atoms.^25^ Our calculations evidence the electron density redistribution toward the S atom (Figure S6), with Bader charges on the S atoms of −0.86 and −0.65 |*e*| in S-in-Fe(110) and S-on-Fe(110), respectively (Table S3). However, the loss of electron density on nearby Fe sites was relatively small, ranging from 0.02 to 0.06 |*e*| compared to the Fe site on the pristine Fe(110) surface. Despite this charge redistribution caused by S atoms, the charge transfer toward the adsorbed TCE molecule was lower by 1 order of magnitude at the S sites compared to the Fe sites (Table S4). This is consistent with a much weaker electronic interaction of the TCE molecule with the S site compared to that with the Fe site. While no considerable charge accumulation can be observed between the adsorbed TCE molecule and the underlying S atom at the S site (Figure 2E), there is a substantial increase in charge density between the C=C bond of TCE and the Fe site (Figure 2F), indicating the formation of C–Fe bonds. The weaker electronic interaction of the S site with TCE is further corroborated by a much smaller DFT energy contribution to the total TCE Δ*E*_ads_ (<5%) compared to the Fe site (>35%) (Table S4). In fact, the TCE Δ*E*_ads_ at the S site can be almost completely attributed to dispersion interactions, implying that S sites are less efficient in electron transfer to the adsorbed contaminants than Fe sites.

The nature and scale of the TCE electronic interactions at both sites can also be observed from the DOS plots. The highest occupied molecular orbitals of TCE (Figure 1B) hybridize upon interaction with S and Fe sites to a different extent, resulting in different shifts and broadening of their bands (Figure 2G,2H). The p orbitals of C and Cl interact much more strongly with the Fe site than with the S site of S-in-Fe(110). This is especially the case of the p_*z*_ orbitals perpendicular to the TCE molecular plane, which results in a complete disappearance of the π_C=C_ bond and stabilization of π nonbonding Cl orbitals at the Fe site due to interactions with the Fe 3d orbitals. Results of our electronic analysis clearly show that TCE is more activated for dechlorination reactions at the Fe sites than at the S sites.

Altogether, our results demonstrate that sulfur intrinsically acts as a catalyst poison on (n)ZVI, whereby surface passivation by the products of iron corrosion in aqueous environments is not considered. This inhibitive effect of sulfur is well-documented for most transition metal catalysts, including iron catalysts used in ammonia synthesis, iron carburization, and Fischer–Tropsch synthesis.^54−57^

### 3.3 Higher S Coverage Leads to a Dramatic Increase in the TCE Dechlorination Barrier

We further investigated the TCE dechlorination at two Fe(110) surface models with higher S coverages that reflect the known optimal S/Fe ratios from experimental data for particles synthesized by the postsulfidation method. An accurate atomic representation of the structure of S-nZVI particles prepared by the one-pot method would be much more challenging to construct due to the presence of various Fe^0^, S^0^, and FeS_*x*_ phases in the whole particle volume and nonuniform particle morphology.^12,16^

The optimal S/Fe mole ratio in S-nZVI particles prepared by the postsulfidation method was found in our previous study to be 0.034.^58^ Considering the measured BET-specific surface area of 32.7 m^2^ g^–1^, the averaged S surface coverage remarkably corresponds (by a factor of 1.25) to the formation of an S monolayer on the nZVI with the same topology as the topmost layer of the most stable (001) plane of mackinawite. This corresponds to 50% coverage of Fe(110) hollow sites by S atoms in a regular fashion. Poorly ordered mackinawite has been found to be the dominant FeS_*x*_ mineral on the surface of S-nZVI prepared by the postsulfidation method.^12,16,22,58,59^ The suppression of H_2_ evolution was observed from the S/Fe mole ratio of 0.016,^58^ which corresponds to half the S coverage relative to the surface of mackinawite. Therefore, Fe(110) surface models with 1/4 and 1/2 monolayer coverage by S atoms, termed S_1/4 ML_–Fe(110) and S_1/2 ML_–Fe(110), were considered.

The geometry optimization resulted in TCE adsorbed at both S-covered surfaces in a horizontal position at a distance between the nearest C and surface S atoms of 3.4 and 3.5 Å, respectively (Figure S7). The geometry of TCE in both adsorption complexes remained practically the same as that in the gas phase (Table S2). The calculated Δ*E*_ads_ values were −45.6 kJ mol^–1^ at the S_1/4 ML_–Fe(110) surface and −53.0 kJ mol^–1^ at the S_1/2 ML_–Fe(110) surface and could be entirely ascribed to dispersion interactions, which is in line with the negligible charge transfer to the adsorbed TCE molecule (Table S4). These adsorption energies were comparable to the Δ*E*_ads_ of TCE at the (001) surface of mackinawite (−48.4 kJ mol^–1^).^46^ The adsorption of TCE at both surfaces was thus less favorable than that at S sites of Fe(110) surfaces doped with a single S atom.

**Figure 3:**
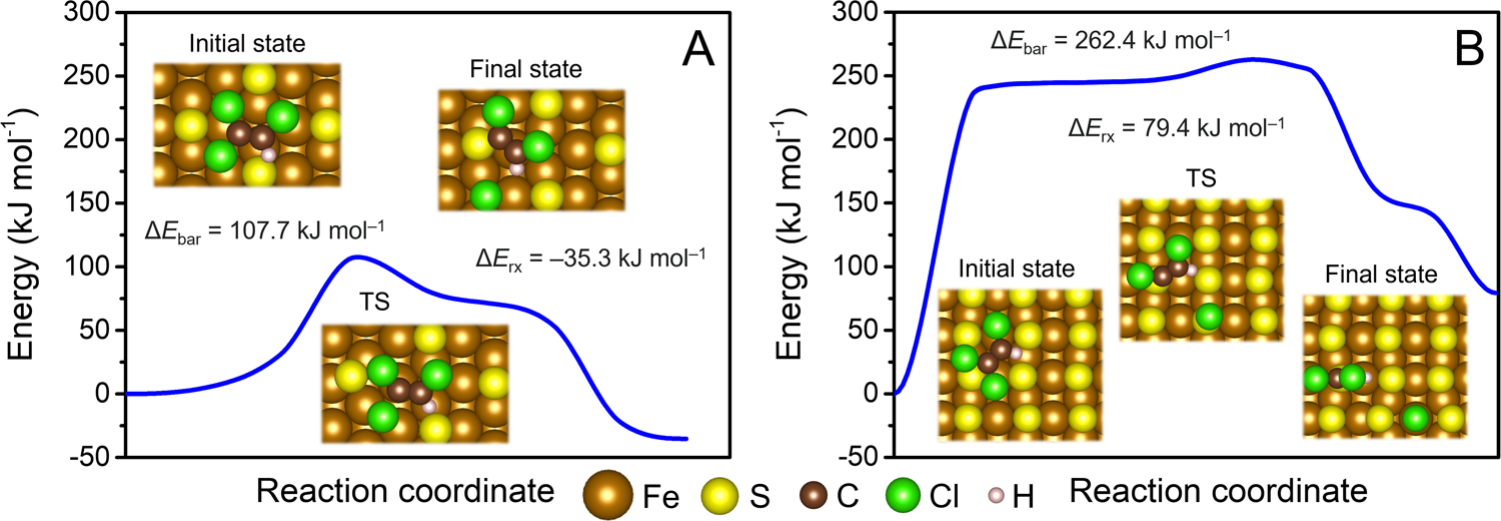
Reaction profiles of TCE dechlorination at the (A) S_1/4 ML_-Fe(110) and (B) S_1/2 ML_-Fe(110) surfaces. Insets show the calculated geometries, and TS denotes the transition state. Only cleavage of the weakest C–Cl bond was calculated as no other Cl atoms spontaneously dissociated during structural relaxations.

Both S_1/4 ML_–Fe(110) and S_1/2 ML_–Fe(110) surfaces also exhibited a high reaction barrier for the rate-limiting C–Cl cleavage of the TCE molecule (Figure 3A,3B), reaching 107.7 and 262.4 kJ mol^–1^, respectively. Such barriers are substantially higher than those calculated at the surfaces of pristine Fe and Fe doped with one S atom. Dissociation of one Cl atom resulted in a release of only 35.3 kJ mol^–1^ at S_1/4 ML_–Fe(110), indicating that the TCE dechlorination at this surface is less thermodynamically favorable than that at surfaces with lower S coverage. In the case of S_1/2 ML_–Fe(110), the cleavage of the C–Cl bond was even accompanied by an increase in the total electronic energy by 79.4 kJ mol^–1^. The products of the first Cl cleavage at both S_1/4 ML_–Fe(110) and S_1/2 ML_–Fe(110) were bound in an upright orientation to the surface via an S atom, being in an unfavorable position for subsequent dechlorination reactions. As a result, no spontaneous dechlorination of other Cl atoms was observed, as opposed to calculations with Fe, S-in-Fe(110), and S-on-Fe(110) surfaces. The TCE dechlorination barrier at the S_1/2 ML_–Fe(110) surface is remarkably close to that at the (001) surface of mackinawite (252.0 kJ mol^–1^).^46^

It has been hypothesized that sulfidation may increase the reactivity of (n)ZVI surface by shifting the Fe 3d band center.^25^ This was based on the principle of the d-band center theory, which postulates that the reactivity of transition metals is correlated to the proximity of their d-band center to the Fermi level.^60^ We calculated the projected DOS of the Fe 3d electrons on Fe(110) surfaces with increasing S coverage (Figure S8). As the degree of sulfidation increases, the d-band centers of the up and down spin states shift to lower energies relative to the Fermi level, leading to a retreat of the valence d-band center from the Fermi level. These results suggest that electron redistribution in (n)ZVI upon sulfidation does not enhance its reactivity with contaminants. This is in line with previous studies that reported a shift of the metal d-band center away from the Fermi level upon sulfidation and, consecutively, inhibition of surface-mediated reactions.^61−64^ Interestingly, there is a correlation between the TCE dechlorination barriers and the centers of the Fe spin-up d-band at Fe surfaces doped to various extents with S atoms (Figure S9). The inhibition of TCE dechlorination, however, cannot be solely ascribed to electronic effects, as S atoms also hinder the accessibility of reactive Fe sites.

It should be noted that the S_1/4 ML_–Fe(110) and S_1/2 ML_–Fe(110) models provide only simplified representations of the S-nZVI surface with the optimal S/Fe ratio. Besides neglecting the presence of iron corrosion products on the particle surface, the role of which is discussed in detail in the following section, they do not account for other Fe facets, steps, kinks, and other surface defects, which could contain more reactive sites for dechlorination reactions. Furthermore, the postsulfidation method does not produce a perfectly uniform monolayer of S atoms, but the S atoms are distributed within a depth of several nm from the particle surface in poorly ordered phases,^58^ resulting in the dilution of S coverage on the particle surface. Nevertheless, the results of our models with higher S coverage are in agreement with the trends observed on the Fe(110) surfaces doped with a single S atom and further illustrate that sulfidation of pristine Fe intrinsically inhibits the dechlorination of TCE. This is also in line with the poor reactivity of FeS_*x*_ minerals mackinawite and pyrite with TCE observed experimentally under anaerobic conditions.^13,65,66^

### 3.4 Sulfidation Passivates the (n)ZVI Surface Less Than Oxidation in Aqueous Media

Reactions of (n)ZVI with contaminants occur typically in aqueous media. Upon contact with water, (n)ZVI rapidly develops a surface passivation layer formed by iron (oxyhydr)oxides.^67^ Owing to the high reactivity of Fe with water, the pristine (n)ZVI surface used as a reference in our calculations is virtually absent on particles when they are applied to ground or surface waters. To investigate the effect of (n)ZVI surface corrosion on its reactivity with TCE at the atomic resolution, we used two Fe(110) surface models doped with (a) one O atom and (b) one OH group. These models were analogous to the S-on-Fe(110) model discussed above.

TCE adsorbed at both the O-on-Fe(110) and OH-on-Fe(110) surfaces in a tilted orientation similar to that at the S-on-Fe(110) surface (Figure S10), with distances of 3.0 and 2.4 Å between the nearer C atom of TCE and the terminal O and H atoms, respectively. In both adsorption complexes, the TCE geometry was only slightly distorted (Table S2). The Δ*E*_ads_ of TCE were −70.2 kJ mol^–1^ at the O-on-Fe(110) surface and −57.1 kJ mol^–1^ at the OH-on-Fe(110) surface and could be entirely attributed to dispersion interactions (Table S4). The more favorable adsorption of TCE at the O-on-Fe(110) surface compared with Fe surfaces doped with OH and S adatoms stems from the smaller sterical hindrance of the O atom.

The high electronegativity of the O atom caused charge redistribution toward the O/OH species to a similar extent as the addition of an S atom (Figure S11), with Bader charges of −0.97 and −0.58 |*e*| on the O atom and the OH group, respectively (Table S3). Similarly to S-doped surfaces, the accumulation of negative charge on the dopant atoms did not result in a substantial electron density loss at nearby Fe sites (Table S3) or in a stronger charge transfer toward the adsorbed TCE molecule (Table S4).

**Figure 4:**
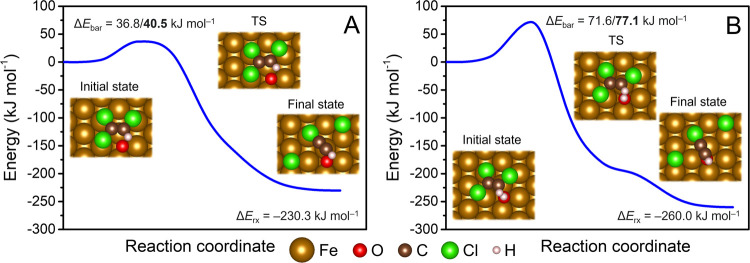
Reaction profiles of TCE dechlorination at the (A) Fe(110) surface doped with a single O atom and (B) Fe(110) surface doped with a single OH group. Values in bold include solvation effects modeled using the continuum solvation model VASPsol. Insets show the calculated geometries, and TS denotes the transition state.

The calculated barriers for the TCE *trans*-*β-*dichloroelimination reaction at the O and OH sites reached 36.8 kJ mol^–1^ and 71.6 kJ mol^–1^, respectively (Figure 4A,4B). The reaction products were oriented similarly as at the S site of S-doped Fe(110), with cleaved Cl atoms at the hollow Fe sites and chloroacetylene remaining close to the original TCE position at a distance of ∼3.3 and ∼2.2 Å from the terminal O atom and OH group, respectively. The released reaction energies were 230.3 and 260.0 kJ mol^–1^, being comparable to the energies released during TCE *trans*-*β*-dichloroelimination at the S sites of Fe(110) surfaces doped with one S atom.

To account for solvation effects, we recalculated dechlorination barriers at the investigated sites using the continuum solvation model VASPsol.^36,37^ This model effectively captures the mean-field interactions between the modeled system and the bulk solvent and provides a dielectric medium that screens electrostatic interactions between charged species. The solvation-corrected TCE dechlorination barriers followed the order: Fe site < S-in-Fe site < S-on-Fe site < O-on-Fe site ≪ OH-on-Fe site (Table 1). While solvation did not substantially alter the reaction barriers in most cases, TCE dechlorination at the S-on-Fe site and at the S_1/2 ML_-Fe surface became more favorable. This can be attributed to the stabilization of the leaving Cl atoms through interactions with the solvent. The solvent-mediated stabilization was more pronounced for these Cl atoms since, in the transition state, they are farther away from the Fe sites compared to reactions at other sites. Overall, the calculated barriers in the implicit solvent indicate that the oxidation of the iron surface hinders TCE dechlorination more than sulfidation.

**Table 1:** PBE+D3-Calculated Energy Barriers of TCE Dechlorination on Various Surfaces and Sites in the Gas Phase (Δ*E*_bar_^gas^) and in the Solvent (Δ*E*_bar_^solv^) in kJ mol^–1^

**surface site**	**Δ*****E***_**bar**_^**gas**^	**Δ*****E***_**bar**_^**solv^a^**^	*N***° of cleaved Cl**
Fe(110)	negligible	negligible	2
Fe(111)	8.8	8.3	3
S-in-Fe(110): S site	27.7	32.7	2
S-in-Fe(110): Fe site	negligible	negligible	2
S-on-Fe(110): S site	51.4	34.9	2
S-on-Fe(110): Fe site	negligible	negligible	2
S_1/4_ _ML_-Fe(110)	107.7	109.3	1
S_1/2__ML_-Fe(110)	262.4	233.3	1
FeS_m_(001)^b^	252.0	235.8	1
O-on-Fe(110): O site	36.8	40.5	2
OH-on-Fe(110): OH site	71.6	77.1	2
H-on-Fe(110): H site	23.6	26.9	2

a Solvation effects were modeled using the continuum solvation model VASPsol.^36,37^

b Energy values for TCE dechlorination at the mackinawite (001) surface are taken from ref.^46^

When pristine nZVI is exposed to water, it predominantly becomes covered with OH groups that originate from the dissociation of water molecules.^68−70^ The OH groups on the Fe surface can be stabilized through mutual hydrogen bonding, leading to a more thermodynamically favorable coverage compared to O atoms, which electronically repel each other.^71,72^ Moreover, preadsorbed O facilitates the dissociation of water molecules at the Fe surface by bonding with an H atom, resulting in two adsorbed OH groups.^71−74^ Indeed, amakinite (Fe(OH)_2_) has been identified as the primary product of iron corrosion in water under anaerobic conditions.^39^ Hence, the surface of freshly oxidized (n)ZVI will be covered mainly by the OH groups, which have a more inhibitive effect on TCE dechlorination than the O adatoms (Table 1). The primary iron corrosion product, amakinite, eventually transforms into more stable iron (oxyhydr)oxides,^39,67^ which together form a several-nm-thick passivating layer.^75^ Considering the known inhibition of (n)ZVI reactivity by its corrosion products,^5,67,76^ it is reasonable to anticipate that the reactivity of these (oxyhydr)oxide phases in TCE dechlorination will be remarkably lower than that of the O-on-Fe(110) and OH-on-Fe(110) models, which contain only one O- and OH-doped site.

It is also noteworthy that the TCE *β*-elimination barriers at the Fe, S, and O/OH sites follow the same trend as the band gaps of metallic iron (0.0 V), iron sulfides (0.0–0.95 eV), and iron (oxyhydr)oxides (typically 2.0–3.0 eV).^77−79^ While band gaps are a good measure of electron transfer resistance in bulk crystalline materials, our calculations reveal the electron transfer characteristics of individual atoms in the topmost surface layer. These approaches provide complementary insights into the electron transfer resistance of poorly crystalline sulfide and (oxyhydr)oxide phases that cover the Fe^0^ core. Both methods indicate that sulfidation does not directly enhance electron transfer compared to metallic Fe^0^ but it passivates the (n)ZVI surface less than oxidation in aqueous environments.

### 3.5 Sulfidation Creates Highly Selective Fe Sites for Electron Transfer toward Contaminants

**Figure 5:**
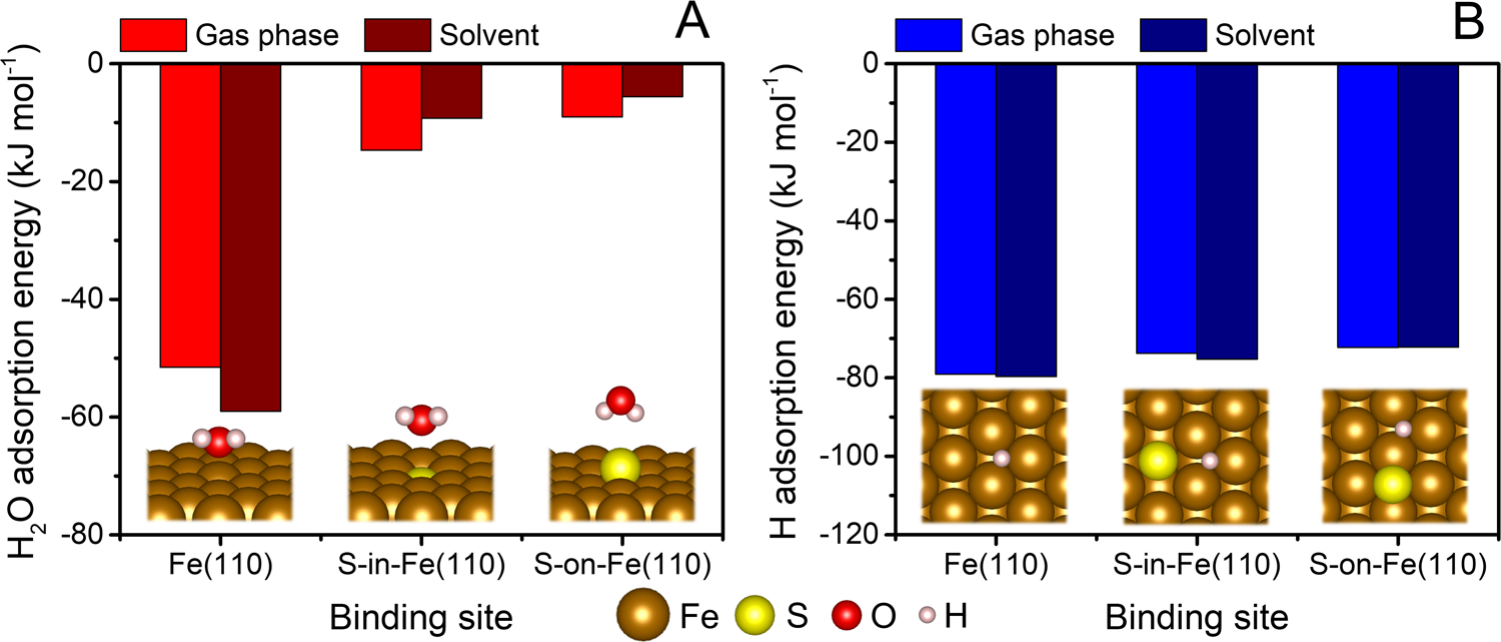
PBE+D3-calculated adsorption energies of (A) water and (B) H* at pristine and S-doped Fe(110) surfaces in the gas phase and solvent. Solvation effects were modeled using the continuum solvation model VASPsol. Insets show the calculated geometries.

S atoms at the (n)ZVI surface have been shown to effectively hinder surface corrosion by preventing the adsorption of water and H* at the S sites and nearby Fe sites.^19,20,22,51^ Our results align with these previous studies, as we observed substantially less favorable water adsorption at the S sites compared to that at the pristine Fe(110) surface (Figure 5A). Furthermore, during all structural relaxations, H* migrated away from the S atom toward nearby Fe hollow sites, where its adsorption was slightly less favored compared to that on the pristine Fe(110) surface (Figure 5B). Given the high mobility of adsorbed H* atoms at Fe surfaces,^71,74^ these findings suggest that H* will preferentially migrate away from S atoms. Consequently, the potential for H*-mediated hydrogenolysis and hydrogenation reactions at these sites may be limited. The presence of preadsorbed H* prevents the chemisorption of TCE and its activation for electron transfer-controlled *β*-elimination, leading to an increase in the reaction barrier (Table 1 and Figure S12). A lower concentration of adsorbed H* at Fe sites in proximity to S atoms can, therefore, enhance their reactivity in *β*-elimination reactions.

This study, in conjunction with the cited literature, provides a comprehensive description of the reactive sites and mechanisms underlying the improved selectivity of S-(n)ZVI in the dechlorination of TCE at the atomic resolution. While sulfidation alone does not intrinsically enhance the reactivity of pristine (n)ZVI with target contaminants as assumed by others,^25^ it generates highly reactive Fe sites that exhibit lower affinity for water adsorption and dissociation, rendering them more hydrophobic than the Fe sites on the pristine Fe surface. The limited interaction of these sites with water effectively suppresses H_2_ evolution and particle corrosion, as previously suggested by Li et al.^20^ Moreover, the less favorable H* adsorption at these Fe sites increases their selectivity for direct electron transfer-mediated reactions with hydrophobic contaminants. While the dechlorination of TCE can potentially also occur directly at the S sites, this reaction is kinetically less favorable compared to Fe sites, which enable more efficient electron transfer.

A potential limitation of this study is our focus on TCE dechlorination reactions controlled by direct electron transfer (i.e., *β*-elimination). The significance of the competing H*-mediated hydrogenolysis has recently sparked debate. Most of the previous studies on TCE dechlorination by S-(n)ZVI concluded that H*-mediated reactions are hindered by sulfidation, which was supported by slower acetylene hydrogenation rates and weaker H* adsorption near S sites.^11,19,51^ Yet, two recent studies have proposed that this pathway could substantially contribute to the reduction of less chlorinated ethenes, such as vinyl chloride and dichloroethene isomers, especially at low S surface coverage.^80,81^ Hence, the effects of sulfidation on H* stability, recombination, and reactivity with contaminants at the (n)ZVI surface warrant further investigation.

## 4 Conclusions

This study characterizes, for the first time, the reactivity of different S-(n)ZVI surface sites with TCE at the atomic scale, providing novel insights into the mechanism underlying the enhanced selectivity of S-(n)ZVI in contaminant removal. By employing molecular modeling techniques based on DFT, we demonstrated that sulfidation intrinsically hinders the TCE dechlorination at pristine (n)ZVI and that its dechlorination-promoting effects only become apparent when the (n)ZVI surface undergoes corrosion. In particular, the S sites on S-(n)ZVI exhibit higher reactivity in TCE *β*-elimination compared to the O and OH sites typically present on the surface of (n)ZVI freshly oxidized in water. Furthermore, the presence of S atoms protects nearby reactive Fe sites, preventing their oxidation and thereby enhancing their availability and selectivity for direct electron transfer-mediated reactions with hydrophobic contaminants. These findings suggest that the performance of S-(n)ZVI in contaminant removal is governed by a delicate interplay between the intrinsic inhibitory effects and the corrosion-protecting properties of the S atoms.

The intrinsic effects of sulfidation described here for the TCE reduction apply to the dehalogenation of other organic contaminants, including halogenated hydrocarbons, pharmaceuticals, and flame retardants. Our findings reveal that sulfidation is unlikely to enhance the (n)ZVI reactivity in gas-phase reactions in the absence of oxidants. However, in typical synthesis and application scenarios where (n)ZVI is prepared through aqueous phase processes and introduced into groundwater, the particle surface is exposed to water and oxygen. Consequently, the corrosion protection afforded by sulfidation will generally outweigh its intrinsic inhibitory effects. Reductive dechlorination through electron transfer will then preferentially occur at Fe sites adjacent to S atoms, given that sulfidation will limit the oxidation and passivation of these sites. However, the accessibility of these reactive Fe sites could be lower for bulkier molecules, such as perchloroethene or brominated hydrocarbons, due to steric hindrance of the nearby S atoms.

A more comprehensive understanding of the intrinsic inhibitory effects of S atoms in the reductive dehalogenation of contaminants at S-(n)ZVI surfaces, while considering different S coverage and active site architectures, would contribute to establishing structure–reactivity relationships facilitating the tailored design of S-(n)ZVI for enhanced contaminant removal. This is of particular significance for chlorinated ethenes as their dechlorination mechanisms, including the role of H*-mediated reactions, remain a subject of debate.

## Data Availability

The data from DFT calculations underlying this study are openly available in Zenodo at https://zenodo.org/record/8311392.

## Supporting Information Available

Computational details; overview of surface slab models and their lattice parameters; configurations of adsorbed TCE with geometric parameters; reaction profile of TCE dechlorination at the Fe(111) surface; charge density redistributions induced by dopants; Bader charges on selected sites; TCE adsorption energies with contributions from pure DFT and D3 dispersion correction; charge transfer from surfaces to adsorbed TCE; projected density of states on Fe surfaces with different extent of sulfidation; relationship between the TCE dechlorination barriers and the position of the Fe d-band center of various surfaces; and effect of preadsorbed H* on TCE adsorption and dechlorination at the Fe(110) surface (PDF)
