# Supplementary material for: Intrinsic Effects of Sulfidation on the Reactivity of Zero-Valent Iron With Trichloroethene: A DFT Study
Source: J Phys Chem C Nanomater Interfaces. 2023 Oct 24;127(43):21063–74. doi: 10.1021/acs.jpcc.3c04459 (PMC10626624; doi:10.1021/acs.jpcc.3c04459)
Supplement: Supplementary file 1 — jp3c04459_si_001.pdf [file jp3c04459_si_001.pdf]

Supporting Information

# Intrinsic Effects of Sulfidation on the Reactivity of Zero-Valent Iron With Trichloroethene: A DFT Study

*Miroslav Brumovský* [a, *], *Daniel Tunega* [a]

[a] University of Natural Resources and Life Sciences, Vienna, Department of Forest- and Soil Sciences, Institute of Soil Research, Peter-Jordan-Straße 82, 1190 Vienna, Austria

* Corresponding author

E-mail address: miroslav.brumovsky@boku.ac.at

<u>Summary</u>

Number of pages: 16

Number of tables: 4

Number of figures: 12

## Text S1. Computational Details

Geometry optimizations of isolated slabs, slabs with adsorbed TCE dechlorination products, and adsorption complexes on Fe(110) slabs with one S/O/OH/H site were performed using the conjugate gradient algorithm as implemented in VASP. To find the most favorable configuration of TCE adsorption complexes on the surfaces of pristine and regularly sulfidated Fe ($S_{1/4\,ML}$-Fe(110) and $S_{1/2\,ML}$-Fe(110) surface models), relaxations were conducted in two steps. First, the TCE molecule was placed ~5 Å above the slabs in different orientations and the initial structures were optimized in internal coordinates with the GADGET code.[1] Subsequently, the preoptimized adsorption complexes were relaxed with the conjugate-gradient algorithm as implemented in VASP. The most energetically favorable adsorption complexes were further considered for the calculation of dechlorination barriers.

The adsorption energy ($\Delta E_{ads}$) of TCE and water molecule was calculated as

$$\Delta E_{ads} = E_{complex} - (E_{surf} + E_{mol}), \quad (1)$$

where $E_{complex}$ is the total electronic energy of the TCE/water molecule adsorbed on the surface, $E_{slab}$ is the total electronic energy of the bare surface, and $E_{mol}$ is the total electronic energy of the isolated TCE/water molecule in the vacuum. A negative $E_{ads}$ corresponds to a stable TCE-surface complex. The adsorption energy of atomic hydrogen was calculated as

$$\Delta E_{ads} = E_{complex} - (E_{surf} + 1/2\ E_{H2}), \quad (2)$$

where $E_{H2}$ is the total electronic energy of an isolated $H_2$ molecule in the vacuum.

Reaction pathways of surface-mediated dechlorination reactions of TCE were determined with the climbing image nudged elastic band (CI-NEB) method.[2] The reaction coordinates were partitioned into nine images and relaxed until the change in the energy barrier was smaller than 0.005 eV. If no transition state appeared, two additional images were added after the first image, and the calculation was repeated. The energy barrier ($\Delta E_{bar}$) was calculated relative to the energy of the reactant ($E_{react}$) as

$$\Delta E_{bar} = E_{TS} - E_{react}, \quad (3)$$

where $E_{TS}$ is the energy of the transition state. The reaction energy ($\Delta E_{rx}$) was calculated as the difference between the total electronic energy of the product ($E_{prod}$) and reactant ($E_{react}$) as

$$\Delta E_{rx} = E_{prod} - E_{react}. \quad (4)$$

Bader charge analysis[3] was performed to understand the charge transfer in doped surfaces and adsorption complexes. Density of states (DOS) calculations were performed using the tetrahedron smearing method with Blöchl corrections.[4] The post-processing of DOS calculations was performed using the program VASPKIT.[5] Solvation effects were included in the calculations by using the continuum solvation model VASPsol[6,7] developed for solid-liquid interfaces and implemented in the VASP program. The VASPsol calculations were performed as single-point calculations on the gas-phase optimized geometries. Visualizations were prepared using VESTA 3.[8]

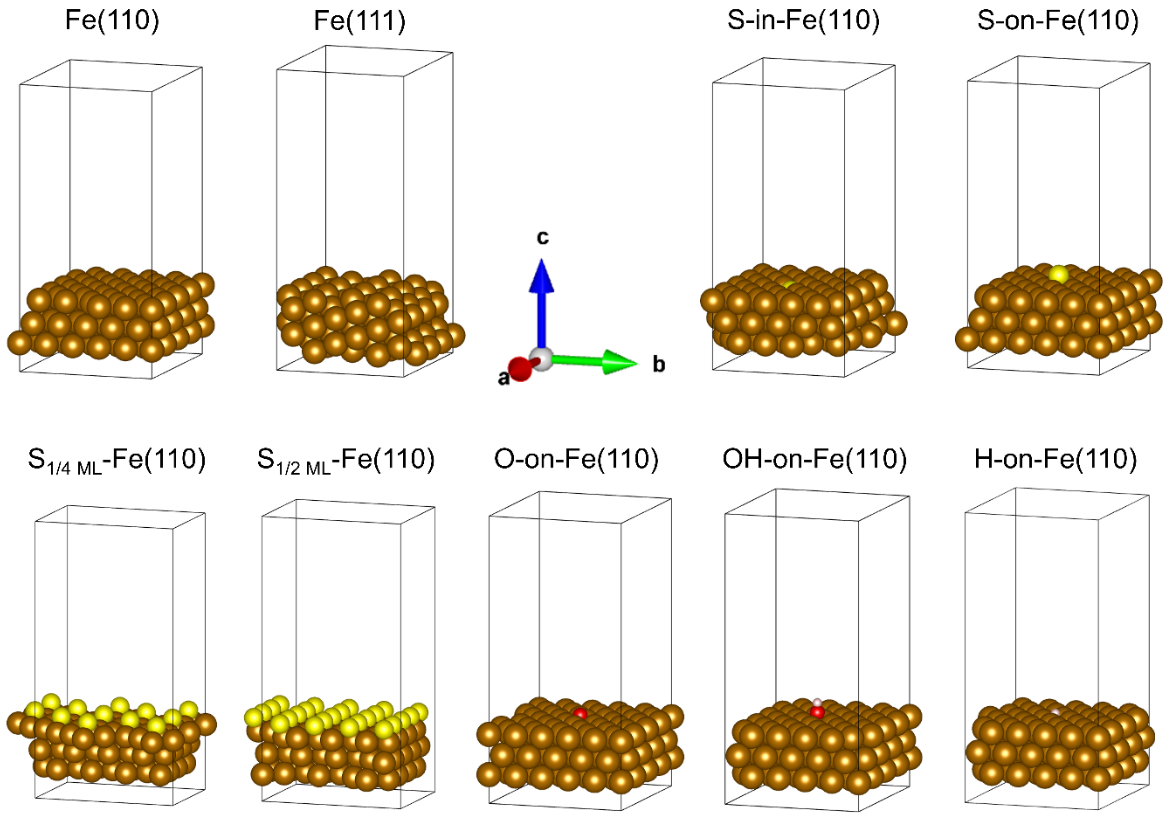


**Figure S1.** Overview of surface slab models used in this study (atom coloring: Fe/brown, S/yellow, O/red, and H/white).

**Table S1.** Lattice parameters of slab models and atomic constraints used in TCE adsorption and dechlorination calculations. Lattice vectors in all slabs were orthogonal (α=β=γ=90°).

| Surface model | Dimensions (Å) | | | Constraints used in TCE adsorption and dechlorination calculations |
|---|---|---|---|---|
| | a | b | c | |
| Fe(110) | 15.881 | 13.889 | 29.031 | All atoms allowed to relax |
| Fe(111) | 15.453 | 13.337 | 30.326 | All atoms allowed to relax |
| S-in-Fe(110) | 15.881 | 13.889 | 29.031 | All atoms allowed to relax |
| S-on-Fe(110) | 15.881 | 13.889 | 29.031 | All atoms allowed to relax |
| $S_{1/4\,ML}$-Fe(110) | 11.124 | 15.789 | 30.551 | Bottom two Fe layers fixed* |
| $S_{1/2\,ML}$-Fe(110) | 11.576 | 15.501 | 30.551 | Bottom two Fe layers fixed* |
| O-on-Fe(110) | 15.881 | 13.889 | 29.031 | All atoms allowed to relax |
| OH-on-Fe(110) | 15.881 | 13.889 | 29.031 | All atoms allowed to relax |
| H-on-Fe(110) | 15.881 | 13.889 | 29.031 | All atoms allowed to relax |

* The bottom Fe layers were fixed to prevent the slabs from collapsing. In all other cases, the slabs were stable during relaxations.

**Table S2.** Geometry of TCE molecule in the gas phase and adsorbed on the modeled surfaces.

| Adsorption complex | Bond length (Å) | | | | | Dihedral angle* (°) | |
|---|---|---|---|---|---|---|---|
| | C=C | C–Cl1† | C–Cl2† | C–Cl3† | C–H | Cl1–C–C–Cl3† | Cl2–C–C–H† |
| *Gas phase TCE* | 1.343 | 1.721 | 1.708 | 1.707 | 1.089 | 180.0 | 180.0 |
| Fe(110)···TCE | 1.485 | 1.721 (fixed) | 1.708 (fixed) | 1.707 (fixed) | 1.113 | 130.8 | 133.0 |
| Fe(111)···TCE | 1.359 | 1.746 | 1.736 | 1.724 | 1.089 | 189.2 | 188.8 |
| S-in-Fe(110): S site···TCE | 1.345 | 1.706 | 1.733 | 1.723 | 1.089 | 187.8 | 185.5 |
| S-in-Fe(110): Fe site···TCE | 1.485 | 1.721 (fixed) | 1.708 (fixed) | 1.707 (fixed) | 1.112 | 131.9 | 133.2 |
| S-on-Fe(110):S site···TCE | 1.343 | 1.704 | 1.743 | 1.713 | 1.089 | 183.9 | 183.2 |
| S-on-Fe(110):Fe site···TCE | 1.487 | 1.721 (fixed) | 1.708 (fixed) | 1.707 (fixed) | 1.126 | 133.8 | 127.4 |
| $S_{1/4\ ML}$-Fe(110)···TCE | 1.344 | 1.720 | 1.708 | 1.705 | 1.088 | 181.0 | 179.6 |
| $S_{1/2\ ML}$-Fe(110)···TCE | 1.344 | 1.721 | 1.709 | 1.707 | 1.089 | 179.1 | 179.7 |
| O-on-Fe(110): O site···TCE | 1.342 | 1.703 | 1.744 | 1.718 | 1.088 | 186.2 | 185.1 |
| OH-on-Fe(110): OH site···TCE | 1.348 | 1.708 | 1.741 | 1.705 | 1.091 | 183.7 | 182.0 |
| H-on-Fe(110): H site···TCE | 1.347 | 1.705 | 1.735 | 1.727 | 1.088 | 185.0 | 184.6 |

* Values <180° indicate the concave orientation of atoms relative to the surface, while values >180° indicate convex orientation.

† Atomic positions:

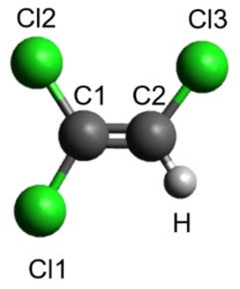

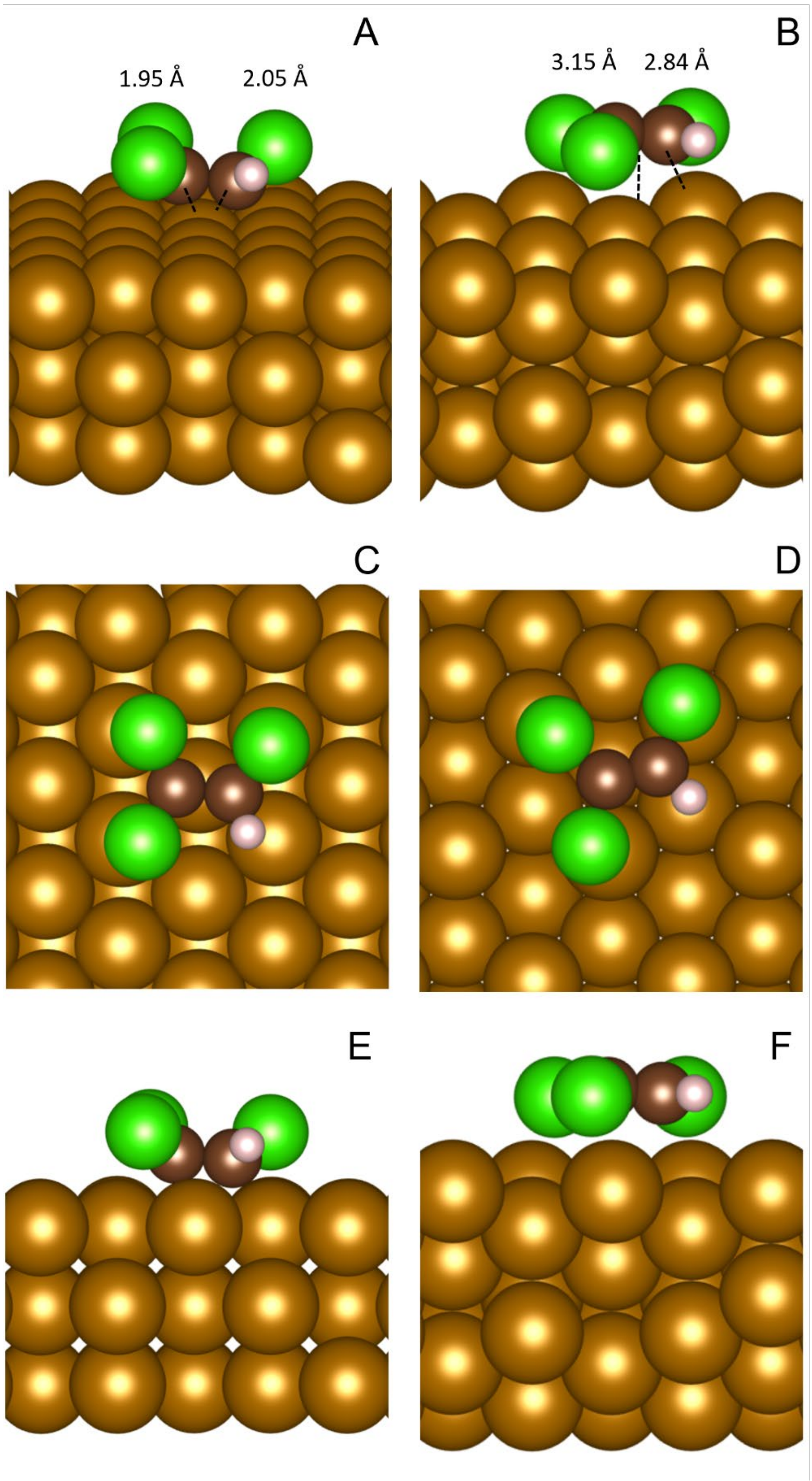


**Figure S2.** PBE+D3-optimized configurations of TCE adsorbed on the Fe(110) (left) and the Fe(111) (right) surfaces: (A, B) overall view with shown C–Fe distances; (C, D) top view; (E, F) side view. Atom coloring: Fe/brown, C/dark brown, Cl/green, and H/white. The TCE adsorption complex on the Fe(110) surface was optimized with frozen C–Cl distances to prevent spontaneous Cl dissociation.

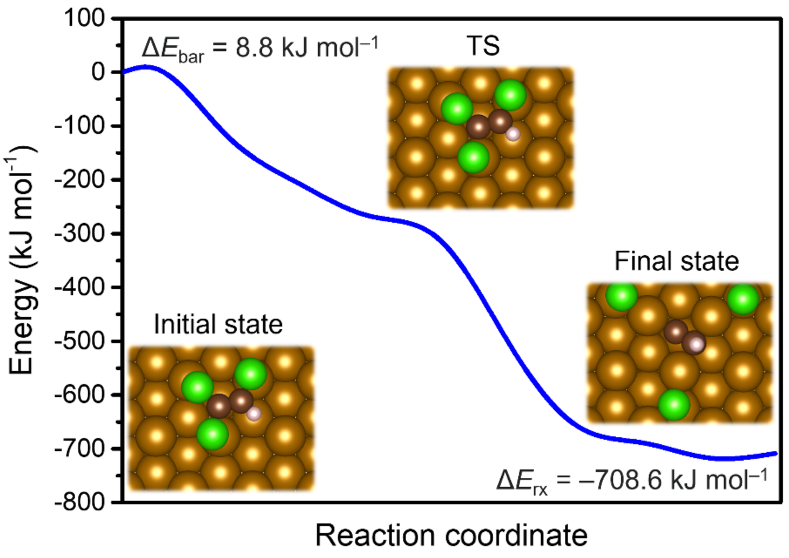


**Figure S3.** Reaction profile of complete TCE dechlorination on the pristine Fe(111) surface. Insets show the calculated geometries and TS denotes the transition state. Atom coloring: Fe/brown, C/dark brown, Cl/green, and H/white.

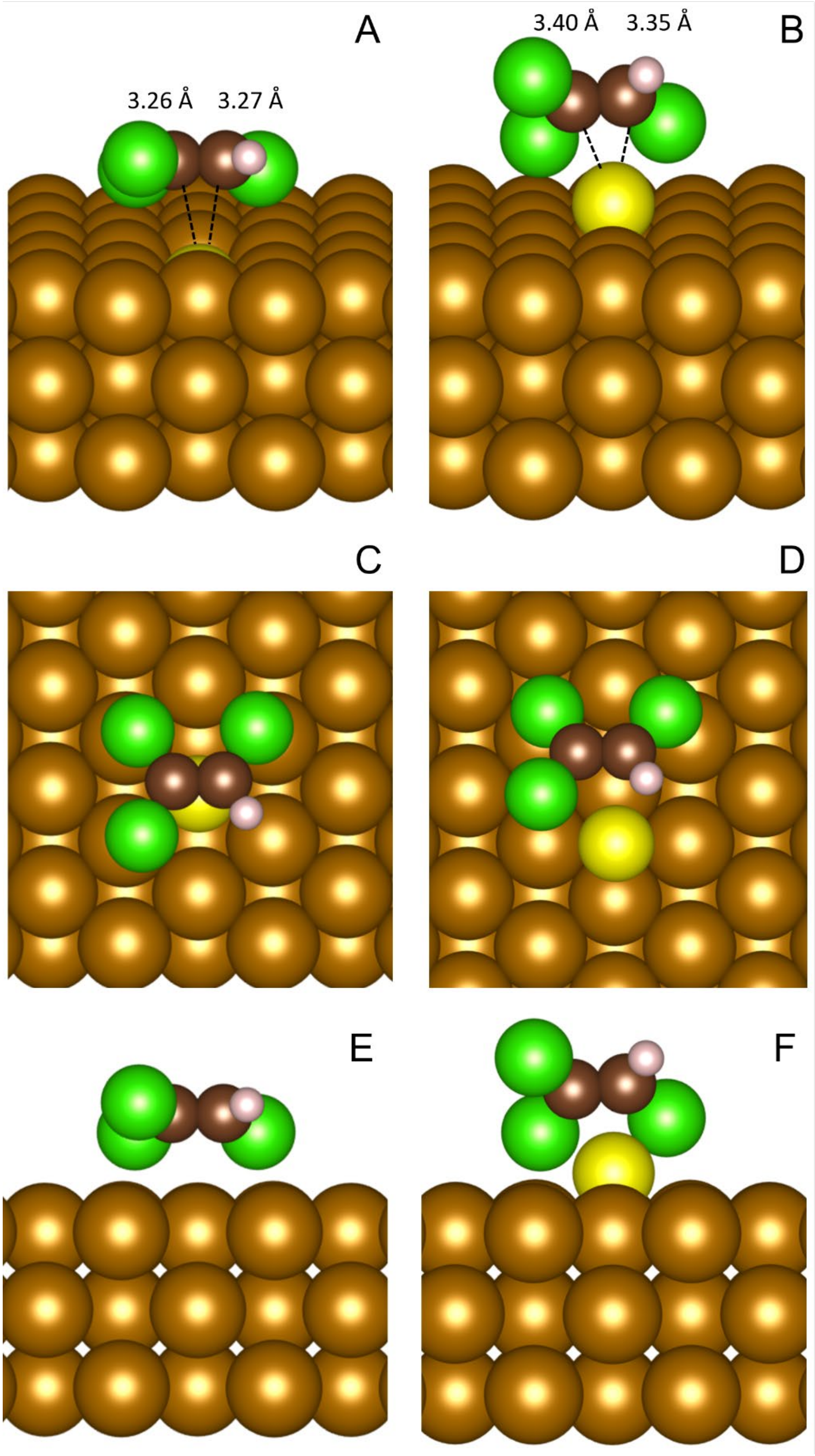


**Figure S4.** PBE+D3-optimized configurations of TCE adsorbed on the S sites of S-in-Fe(110) (left) and S-on-Fe(110) (right) surfaces: (A, B) overall view with shown C–S distances; (C, D) top view; (E, F) side view. Atom coloring: Fe/brown, S/yellow, C/dark brown, Cl/green, and H/white.

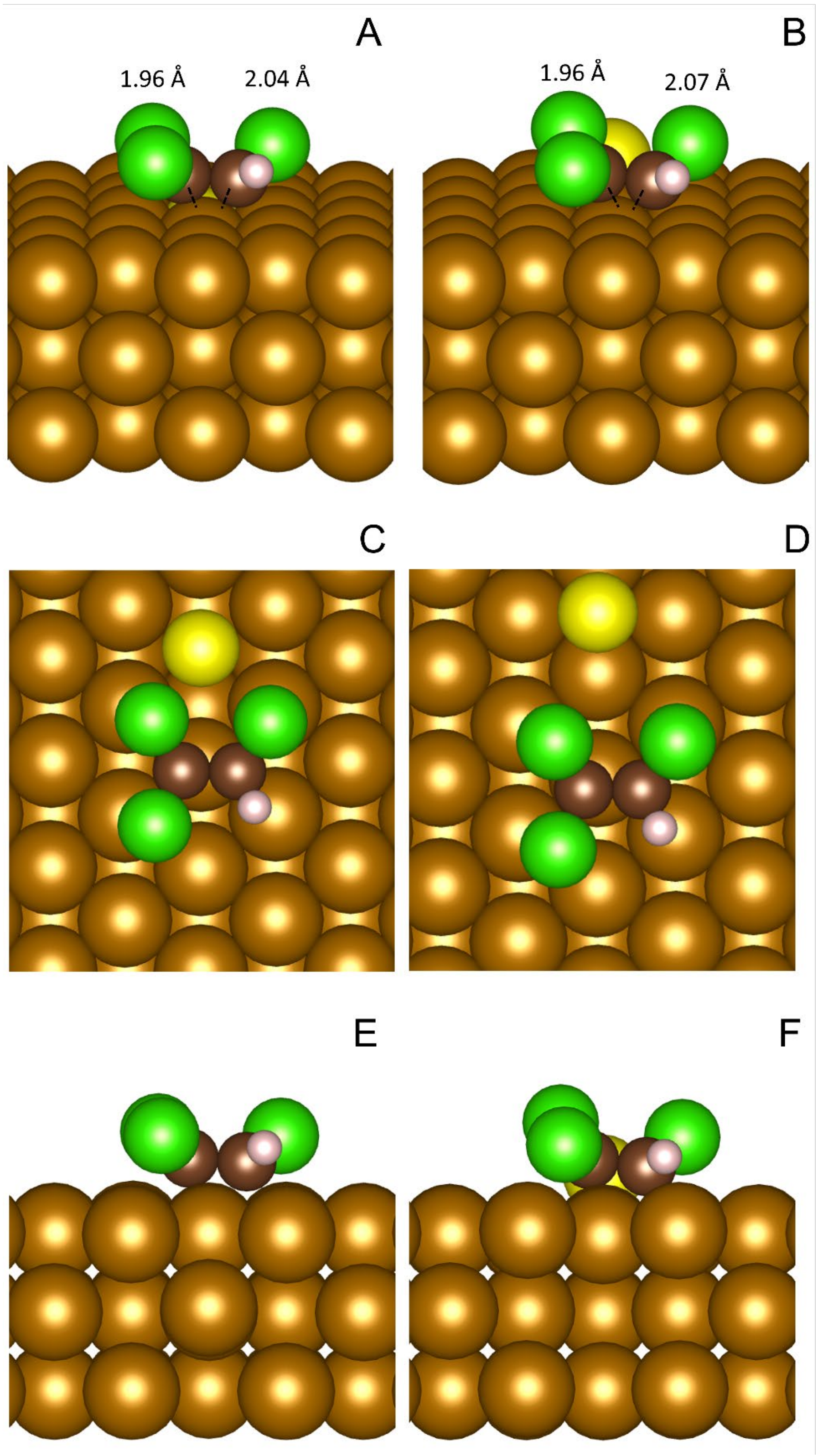


**Figure S5.** PBE+D3-optimized configurations of TCE adsorbed on the Fe sites of S-in-Fe(110) (left) and S-on-Fe(110) (right) surfaces: (A, B) overall view with shown C–Fe distances; (C, D) top view; (E, F) side view. Atom coloring: Fe/brown, S/yellow, C/dark brown, Cl/green, and H/white. The TCE adsorption complexes were optimized with frozen C–Cl distances to prevent spontaneous Cl dissociation.

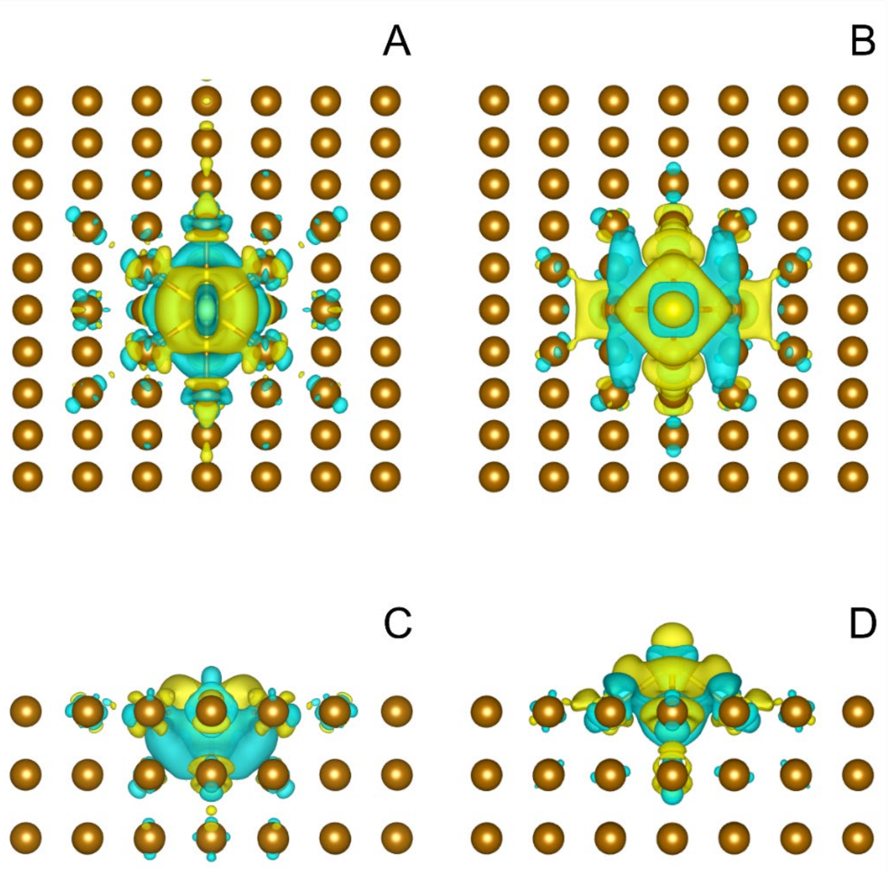


**Figure S6.** PBE-calculated charge density redistribution on the Fe(110) surface induced by a single S atom: (A, C) S-in-Fe(110), (B, D) S-on-Fe(110). The two images of each surface show top and side views, respectively. The yellow isosurface indicates an electron gain, while the blue one represents an electron loss. The isosurface level was set to 0.001 $Bohr^{-3}$.

**Table S3.** Bader charges on atoms at TCE dechlorination sites of selected slab models (no adsorbate present).

| **Surface site** | **Charge in \|*e*\|** |
|---|---|
| Fe(110): Fe site | –0.015 |
| S-in-Fe(110): S site | –0.864 |
| S-in-Fe(110): Fe site | 0.044 |
| S-on-Fe(110): S site | –0.650 |
| S-on-Fe(110): Fe site | 0.007 |
| O-on-Fe(110): O site | –0.974 |
| O-on-Fe(110): Fe site* | 0.006 |
| OH-on-Fe(110): OH site | –1.201 (O), 0.620 (H) |
| OH-on-Fe(110): Fe site* | 0.001 |
| H-on-Fe(110): H site | –0.342 |

* Position of the Fe site was the same relative to the adatoms as in S-on-Fe(110).

**Table S4.** Charge-transfer from slab to the adsorbed TCE molecule calculated with Bader charges in |*e*| and contributions from pure DFT (PBE functional, $\Delta E_{\mathrm{ads}}^{\mathrm{DFT}}$) and empirical dispersion correction (D3, $\Delta E_{\mathrm{ads}}^{\mathrm{disp}}$) to the total TCE adsorption energy ($\Delta E_{\mathrm{ads}} = \Delta E_{\mathrm{ads}}^{\mathrm{DFT}} + \Delta E_{\mathrm{ads}}^{\mathrm{disp}}$) on the modeled surfaces and sites in kJ mol$^{-1}$.

| **Adsorption complex** | **Charge transfer from slab to TCE molecule** | $\boldsymbol{\Delta E_{\mathrm{ads}}}$ | $\boldsymbol{\Delta E_{\mathrm{ads}}^{\mathrm{DFT}}}$ | $\boldsymbol{\Delta E_{\mathrm{ads}}^{\mathrm{disp}}}$ |
|---|---|---|---|---|
| Fe(110)···TCE | 0.650 | –165.6 | –68.8 | –96.8 |
| Fe(111)···TCE | 0.250 | –59.7 | –18.6 | –41.1 |
| S-in-Fe(110): S site···TCE | 0.068 | –81.8 | –3.4 | –78.3 |
| S-in-Fe(110): Fe site···TCE | 0.645 | –166.1 | –68.4 | –97.6 |
| S-on-Fe(110): S site···TCE | 0.059 | –62.8 | 3.2 | –66.0 |
| S-on-Fe(110): Fe site···TCE | 0.663 | –158.8 | –58.4 | –100.4 |
| $S_{1/4\,ML}$-Fe(110)···TCE | 0.002 | –45.6 | 7.3 | –52.8 |
| $S_{1/2\,ML}$-Fe(110)···TCE | 0.005 | –53.0 | 3.0 | –56.0 |
| $FeS_m$(001)···TCE* | Not calculated | –48.4 | 1.8 | –50.2 |
| O-on-Fe(110): O site···TCE | 0.051 | –70.2 | 1.0 | –71.2 |
| OH-on-Fe(110): OH site···TCE | 0.067 | –57.1 | 3.0 | –60.1 |
| H-on-Fe(110): H site···TCE | 0.082 | –71.2 | 5.6 | –76.8 |

* Energy values for TCE adsorption at the mackinawite (001) surface are taken from ref.[9]

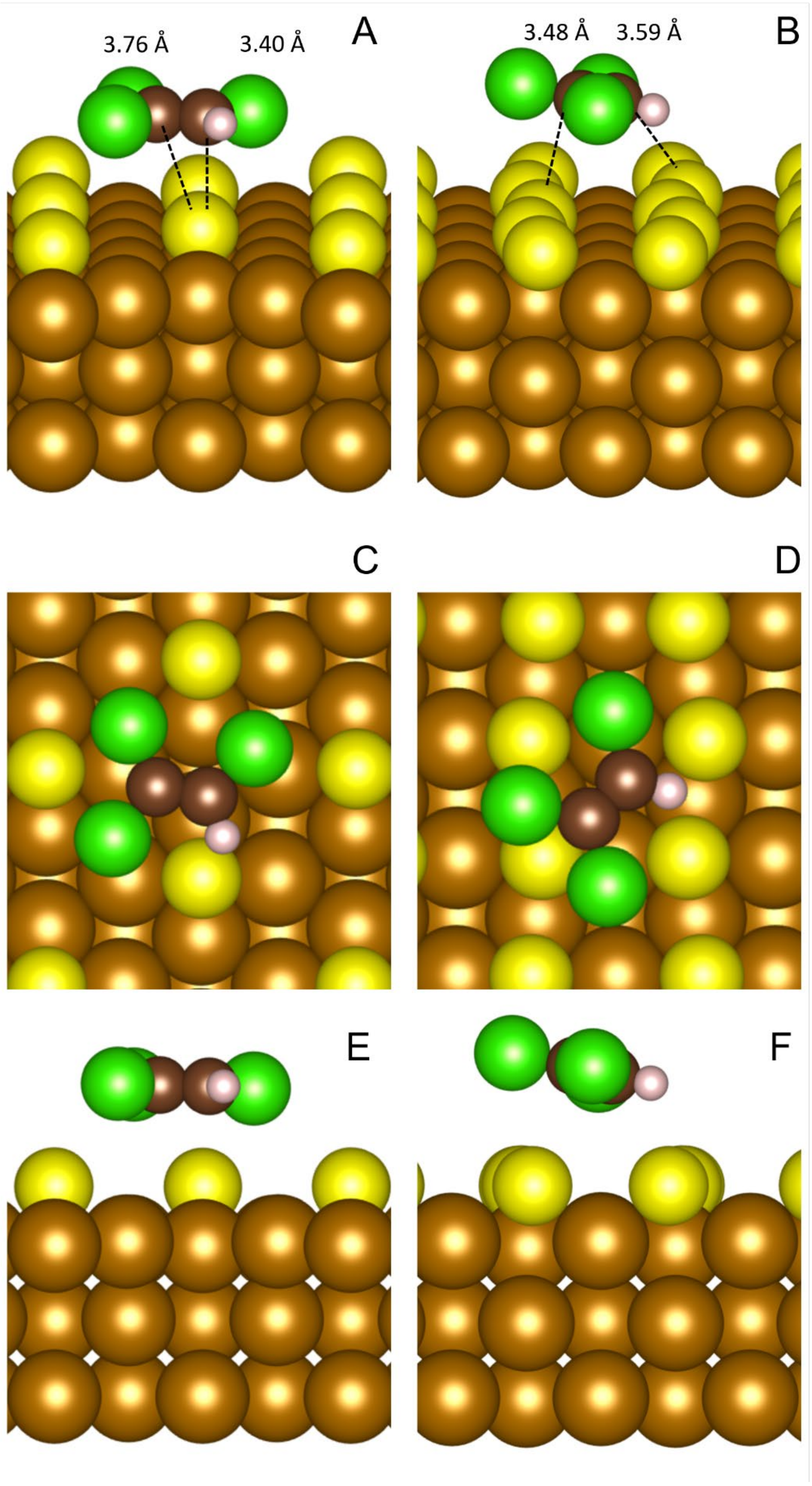


**Figure S7.** PBE+D3-optimized configurations of TCE adsorbed on the $S_{1/4\ ML}$-Fe(110) (left) and $S_{1/2\ ML}$-Fe(110) (right) surfaces: (A, B) overall view with shown C–S distances; (C, D) top view; (E, F) side view. Atom coloring: Fe/brown, S/yellow, C/dark brown, Cl/green, and H/white.

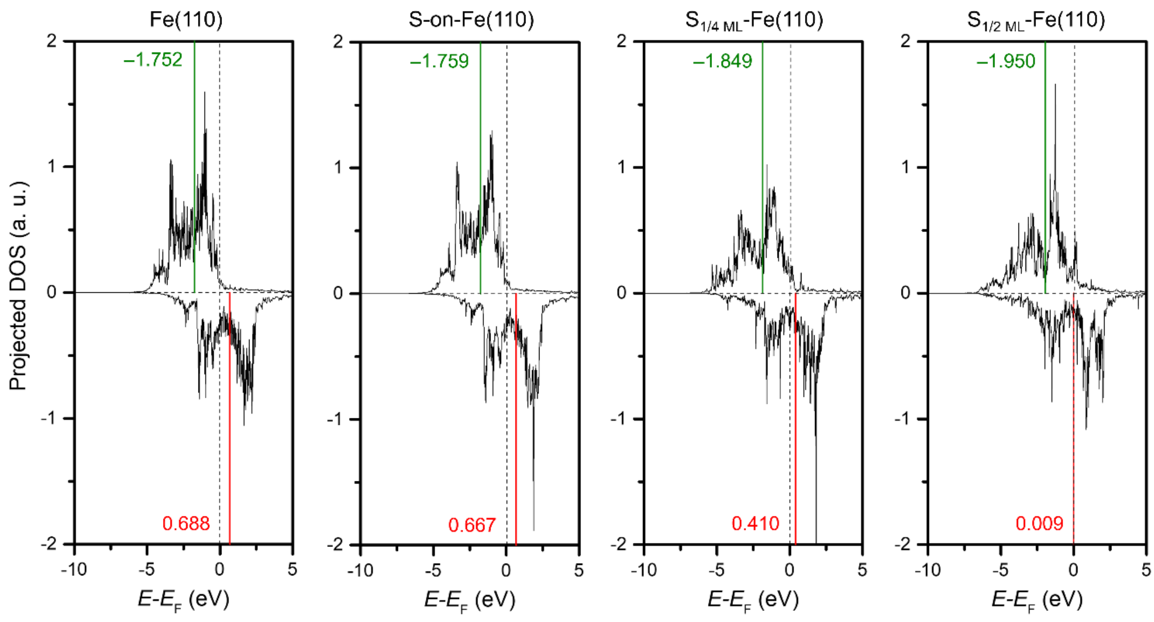


**Figure S8.** Projected density of electronic states of the topmost Fe 3d electrons on the Fe surfaces doped with an increasing number of S atoms (no adsorbate present). The positions of the d-band centers for spin up and down states are indicated in green and red, respectively. The d-band centers are referenced to the Fermi level ($E_F$ = 0 eV).

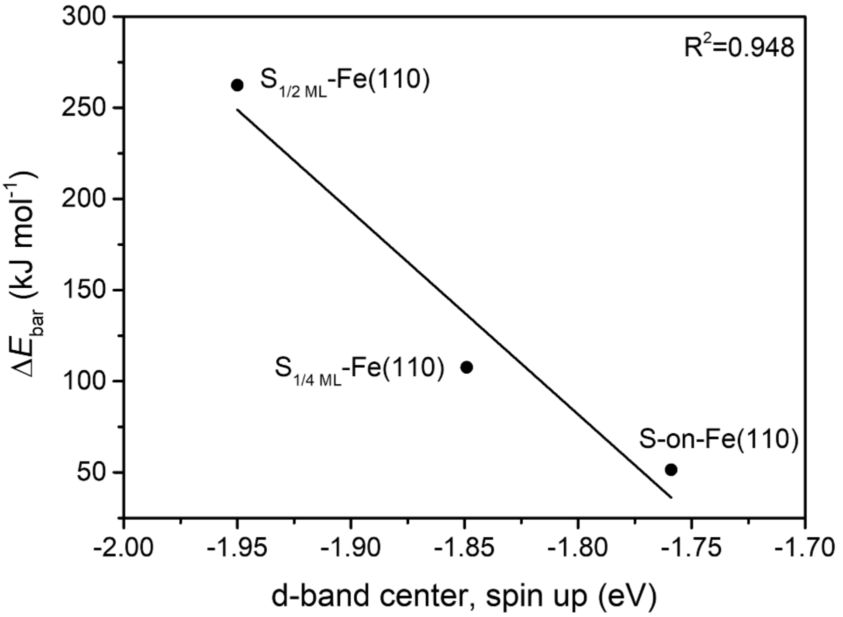


**Figure S9.** Relationship between the TCE dechlorination barriers and the position of the Fe spin up d-band center for surfaces with various extents of sulfidation without adsorbates.

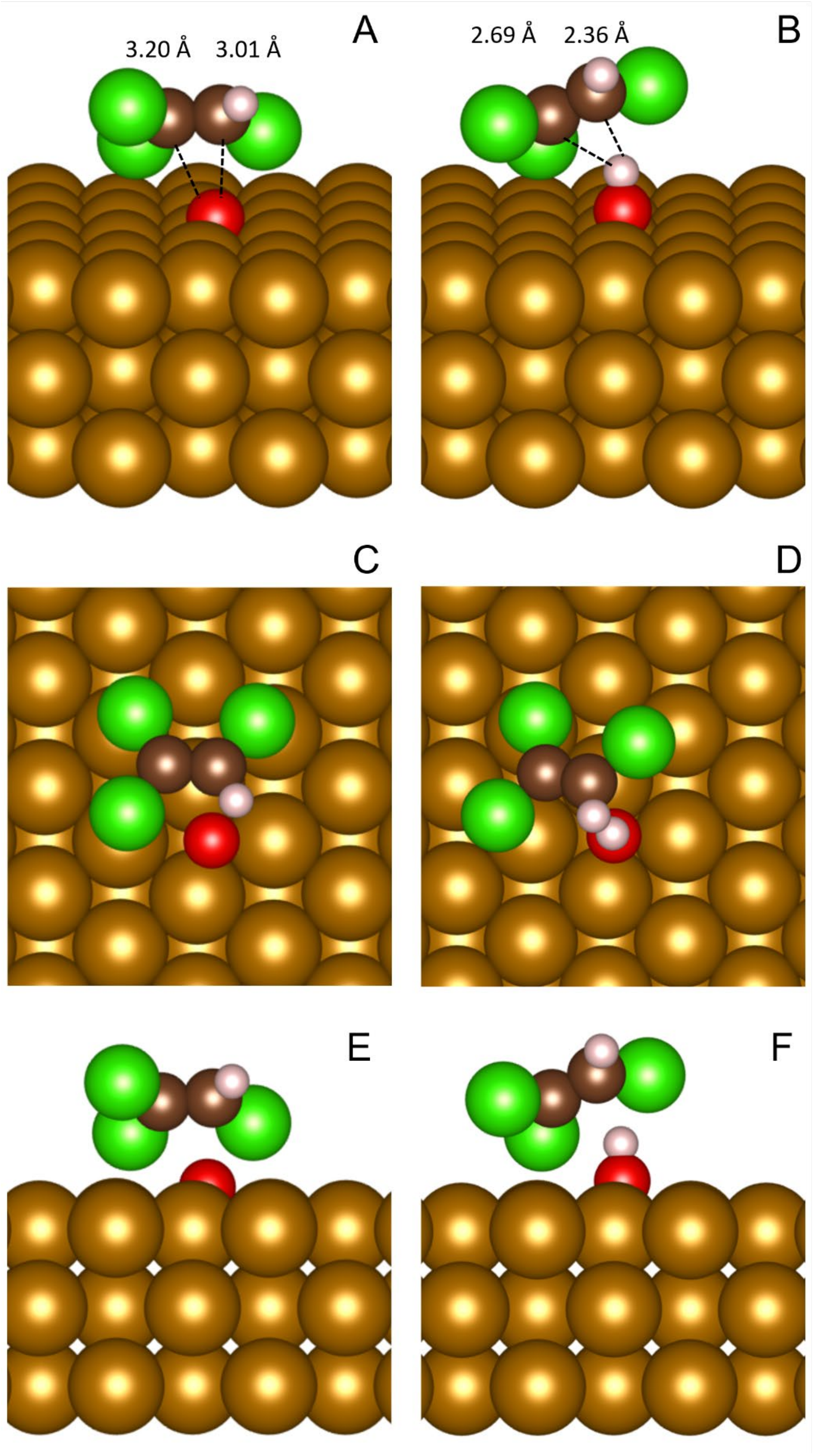


**Figure S10.** PBE+D3-optimized configurations of TCE adsorbed on the O site of O-on-Fe(110) (left) and the OH site of OH-on-Fe(110) (right) surfaces: (A, B) overall view with shown C–O and C–H distances; (C, D) top view; (E, F) side view. Atom coloring: Fe/brown, O/red, C/dark brown, Cl/green, and H/white.

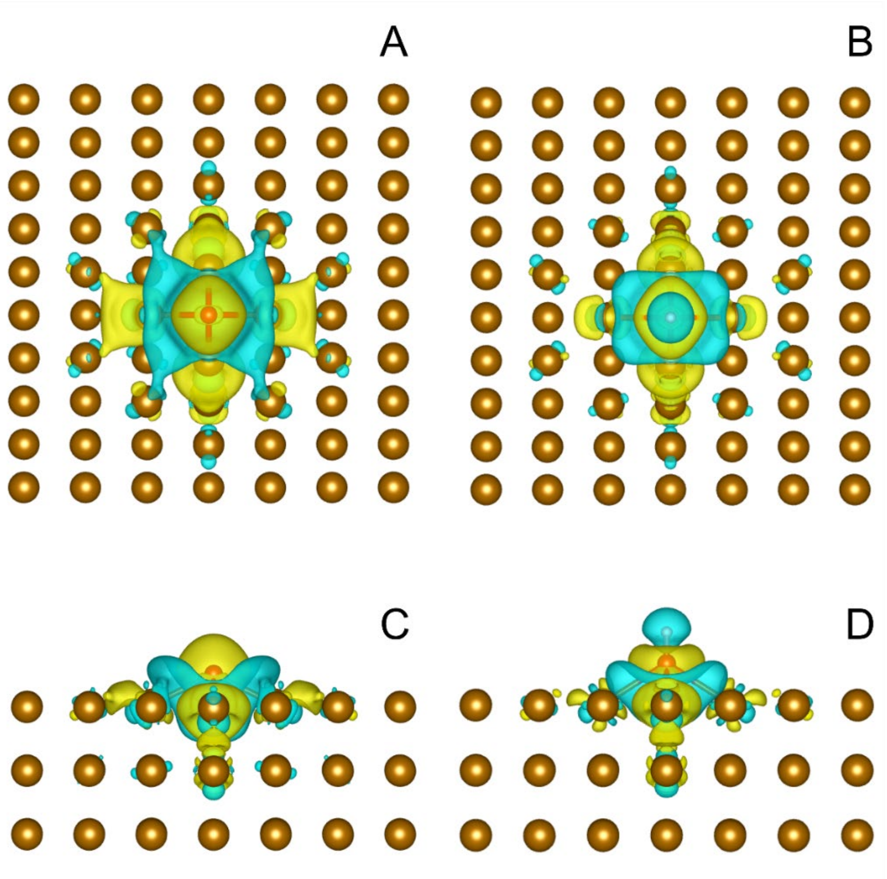


**Figure S11.** PBE-calculated charge density redistribution on the Fe(110) surface induced by a single O atom (A, C) and a single OH group (B, D). The two images of each surface show top and side views, respectively. The yellow isosurface indicates an electron gain, while the blue one represents an electron loss. The isosurface level was set to 0.001 $Bohr^{-3}$.

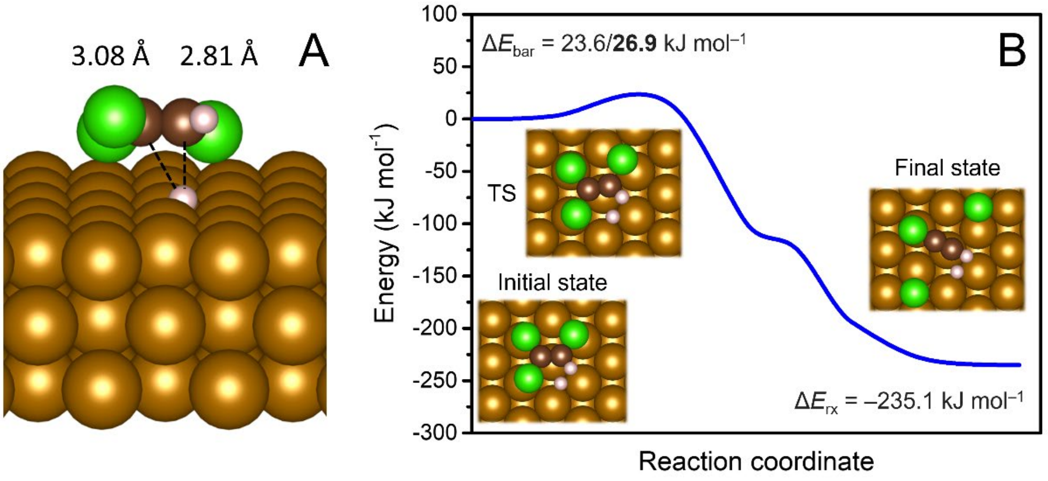


**Figure S12.** TCE dechlorination at the Fe(110) surface with a preadsorbed H* atom: (A) PBE+D3-optimized configuration of TCE adsorbed over the H* atom with shown C–H distances, (B) reaction profile of TCE *trans-β*-elimination. Atom coloring: Fe/brown, C/dark brown, Cl/green, and H/white. Insets show the calculated geometries and TS denotes the transition state. The energy barrier value in bold includes solvation effects modeled using the continuum solvation model VASPsol.

*Comments to Figure S12*

The presence of a preadsorbed H* atom on the Fe(110) surface (referred to as the "H-on-Fe(110)" site) prevented TCE from chemisorbing onto the adjacent Fe atom. Similar to other adatom sites explored in this study, TCE adsorbed at the H-on-Fe(110) site in a tilted orientation with only slightly distorted molecular geometry compared to the gas phase (Table S2). The distances between the C atoms of TCE and the H* atom measured 3.1 and 2.8 Å. The TCE adsorption energy at this site was –71.2 kJ mol$^{-1}$, indicating a weaker interaction than that observed on the pristine Fe(110) surface.

The limited C–Fe interaction and poor activation of the TCE molecule for direct electron-transfer-controlled dechlorination at the H-on-Fe(110) site led to an increase in the TCE *trans-β*-elimination barrier to 23.6 kJ mol$^{-1}$ (26.9 kJ mol$^{-1}$ when including solvation effects). The positions of reaction products were similar to those observed at other adatom sites, with cleaved Cl atoms at the hollow Fe sites and chloroacetylene remaining close to the original TCE position with the nearest C–H distance of 2.7 Å. The released reaction energy of 235.1 kJ mol$^{-1}$ was also in line with TCE dechlorination at other adatom sites. The calculated TCE adsorption energy and *β*-elimination barrier collectively indicate a suppressing effect of preadsorbed H* atoms on the direct electron transfer from the (n)ZVI surface to adsorbed contaminants. While H* could potentially participate in TCE dechlorination through the hydrogenolysis pathway, it was beyond the scope of this study to characterize the effects of sulfidation on H*-mediated reactions.

## References


(1) Bučko, T.; Hafner, J.; Ángyán, J. G. Geometry Optimization of Periodic Systems Using Internal Coordinates. *J. Chem. Phys.* **2005**, *122* (12), 124508. https://doi.org/10.1063/1.1864932.

(2) Henkelman, G.; Uberuaga, B. P.; Jónsson, H. A Climbing Image Nudged Elastic Band Method for Finding Saddle Points and Minimum Energy Paths. *J. Chem. Phys.* **2000**, *113* (22), 9901–9904. https://doi.org/10.1063/1.1329672.

(3) Tang, W.; Sanville, E.; Henkelman, G. A Grid-Based Bader Analysis Algorithm without Lattice Bias. *J. Phys. Condens. Matter* **2009**, *21* (8), 084204. https://doi.org/10.1088/0953-8984/21/8/084204.

(4) Blöchl, P. E.; Jepsen, O.; Andersen, O. K. Improved Tetrahedron Method for Brillouin-Zone Integrations. *Phys. Rev. B* **1994**, *49* (23), 16223–16233. https://doi.org/10.1103/PhysRevB.49.16223.

(5) Wang, V.; Xu, N.; Liu, J.-C.; Tang, G.; Geng, W.-T. VASPKIT: A User-Friendly Interface Facilitating High-Throughput Computing and Analysis Using VASP Code. *Comput. Phys. Commun.* **2021**, *267*, 108033. https://doi.org/10.1016/j.cpc.2021.108033.

(6) Mathew, K.; Sundararaman, R.; Letchworth-Weaver, K.; Arias, T. A.; Hennig, R. G. Implicit Solvation Model for Density-Functional Study of Nanocrystal Surfaces and Reaction Pathways. *J. Chem. Phys.* **2014**, *140* (8), 084106. https://doi.org/10.1063/1.4865107.

(7) Mathew, K.; Kolluru, V. S. C.; Mula, S.; Steinmann, S. N.; Hennig, R. G. Implicit Self-Consistent Electrolyte Model in Plane-Wave Density-Functional Theory. *J. Chem. Phys.* **2019**, *151* (23), 234101. https://doi.org/10.1063/1.5132354.

(8) Momma, K.; Izumi, F. VESTA 3 for Three-Dimensional Visualization of Crystal, Volumetric and Morphology Data. *J. Appl. Crystallogr.* **2011**, *44* (6), 1272–1276. https://doi.org/10.1107/S0021889811038970.

(9) Brumovský, M.; Micić, V.; Oborná, J.; Filip, J.; Hofmann, T.; Tunega, D. Iron Nitride Nanoparticles for Rapid Dechlorination of Mixed Chlorinated Ethene Contamination. *J. Hazard. Mater.* **2023**, *442*, 129988. https://doi.org/10.1016/j.jhazmat.2022.129988.
